# Injectable Hydrogel with Rapid Coagulation, Low Swelling, and High Burst Pressure Tolerance Properties for Long‐Term Management of Bleeding Wound

**DOI:** 10.1002/advs.75329

**Published:** 2026-04-24

**Authors:** Yang Ouyang, Wenfeng Qiu, Weiwen Liang, Xin Zhang, Weijie Liu, Zixin Chen, Yifei Li, Hui Wang, Youchen Tang, Min Li, Rongkang Huang, Zijian Chen, Binghua Ma

**Affiliations:** ^1^ Colorectal Surgery Unit III Guangdong Institute of Gastroenterology Biomedical Innovation Center Key Laboratory of Human Microbiome and Chronic Diseases Sun Yat‐sen University Ministry of Education Guangdong Provincial Key Laboratory of Colorectal and Pelvic Floor Diseases The Sixth Affiliated Hospital Sun Yat‐sen University Guangzhou P. R. China; ^2^ Colorectal Surgery Unit V Guangdong Institute of Gastroenterology Biomedical Innovation Center Key Laboratory of Human Microbiome and Chronic Diseases Sun Yat‐sen University Ministry of Education Guangdong Provincial Key Laboratory of Colorectal and Pelvic Floor Diseases The Sixth Affiliated Hospital Sun Yat‐sen University Guangzhou P. R. China; ^3^ Department of General Surgery (Thyroid Surgery) Guangdong Provincial Key Laboratory of Malignant Tumor Epigenetics and Gene Regulation Medical Research Center Sun Yat‐Sen Memorial Hospital Sun Yat‐Sen University Guangzhou P. R. China; ^4^ Naval Medical Service Training Base Naval Medical University Shanghai P. R. China; ^5^ PCFM Lab School of Chemistry Sun Yat‐sen University Guangzhou P. R. China; ^6^ The Eighth Affiliated Hospital Sun Yat‐sen University Shenzhen P. R. China; ^7^ Department of Gastrointestinal Surgery The Affiliated Dongguan Songshan Lake Central Hospital Guangdong Medical University Dongguan P. R. China; ^8^ Translational Medicine Research Center Naval Medical University Shanghai P. R. China

**Keywords:** anti‐swelling, burst pressure tolerance, hemostasis, injectable hydrogel, long‐term management

## Abstract

Injectable wet‐adhesive hydrogels represent a promising approach for managing traumatic hemorrhage, yet their clinical translation is often hindered by excessive swelling and inadequate sealing stability. Herein, we report an injectable hydrogel (denoted as PACmC) with rapid coagulation, ultrahigh burst pressure tolerance, and low swelling properties for emergency hemostasis and long‐term wound management. PACmC hydrogel is formed by electrostatic self‐assembly of aminated carboxymethyl chitosan (ACmCS) into nanoparticles, which are then crosslinked with NHS‐activated tetra‐arm polyethylene glycol (Tetra‐PEG‐SS) via rapid NHS‐amine coupling, yielding gelation within 22.3 s. The positively charged ACmCS nanoparticles actively aggregate blood components to accelerate hemostasis, while their densely packed network physically restricts water infiltration, resulting in low swelling ratio of only 49.2% within 7 d. For tissue adhesion, the NHS‐ester groups on Tetra‐PEG‐SS covalently bond with amino groups on the wound surface, and ACmCS nanoparticles serve as multivalent crosslinking centers that enhance cohesive strength, together enabling robust wet‐tissue adhesion (46.5 kPa) and an ultrahigh burst pressure tolerance of 701 mm Hg. In rabbit and porcine models of hepatic and splenic hemorrhage, PACmC hydrogel achieves rapid hemostasis and maintains long‐term sealing compared to clinical hemostats (thrombin powder and gauze), providing a new direction in developing novel hemostatic materials.

## Graphical abstract

An injectable PACmC hydrogel with “rigid island‐flexible chain” architecture is developed, featuring low swelling (49.2% within 7 d), strong wet adhesion (46.5 kPa), and high burst pressure (701 mm Hg), which can achieve rapid hemostasis and long‐term sealing in rabbit and porcine models of hepatic and splenic hemorrhage.

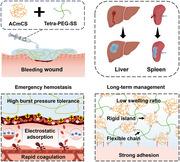

## 1 Introduction

Uncontrolled hemorrhage from intra‐abdominal organs remains a leading cause of trauma‐related mortality [1]. The wet, dynamic, and irregular nature of the wound surfaces, coupled with rapid and voluminous bleeding, imposes stringent demands on hemostatic materials [2]. Conventional hemostatic materials, such as gauze, thrombin lyophilized powder, gelatin sponge, and Fibrin gel, are effective for superficial regular wounds with minor bleeding [3, 4]. However, most hemostatic materials are difficult to effectively control life‐threatening bleeding from abdominal organs due to their inherent limitations, such as insufficient adhesion in wet conditions, weak mechanical properties, and susceptibility to detachment or rapid degradation in dynamic physiological environments [3, 5]. Therefore, the development of advanced hemostatic materials for rapid and effective treatment of uncontrollable bleeding is an urgent need in the fields of trauma care.

Injectable wet‐adhesive hydrogels show unique potential for emergency hemostasis of irregular wounds in intra‐abdominal organs, thanks to their shape adaptability and bio‐adhesive properties [6, 7, 8, 9]. However, these hydrogels still face several challenging limitations. First, most hydrogels suffer from insufficient adhesion for reliable sealing of bleeding sites. Although incorporating multiple adhesive components can partially improve adhesion, these strategies compromise the injectability and biocompatibility of hydrogels [10]. Second, the excessive swelling of hydrogels in wet environment remains a key bottleneck. It often leads to weakened adhesion and mechanical properties, resulting in hydrogel detachment or rupture in vivo and failure to achieve long‐term retention. Common approaches to control swelling include increasing crosslinking density and adding hydrophobic components to hinder water infiltration to stabilize hydrogel network [11, 12]. Nevertheless, higher crosslinking density tends to make hydrogels more brittle, rendering them unsuitable for dynamic physiological conditions [13]. Meanwhile, common approaches to control swelling, such as incorporating hydrophobic components, involve a trade‐off. While incorporating hydrophobic components effectively displaces interfacial water to facilitate adhesion, it can also hinder direct bonding between adhesive groups and tissue interfaces if not carefully balanced. As a result, achieving synergy between strong adhesion and anti‐swelling performance remains challenging [14, 15, 16]. In addition, many strategies aimed at enhancing adhesion and suppressing swelling tend to overlook or even sacrifice the fundamental procoagulant activity essential for immediate hemostasis. Therefore, the construction of a novel in situ fast‐gelling hydrogel that integrates the properties of robust occlusive adhesion, outstanding anti‐swelling performance, and inherent rapid coagulation capability to enable long‐term sealing is expected to significantly accelerate the tissue repair of trauma wounds but remains a huge challenge.

**FIGURE 1:**
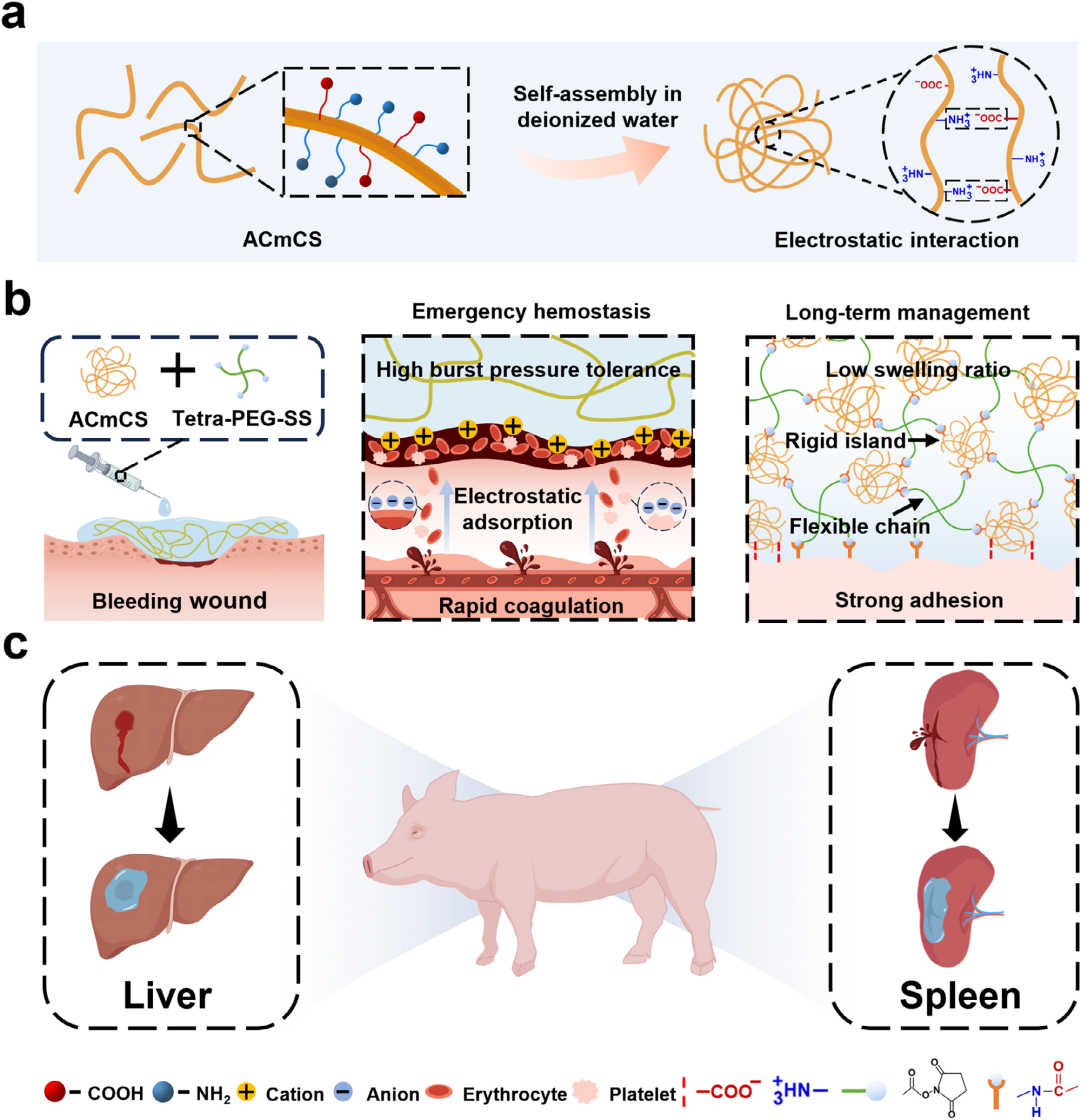
Design and application of PACmC hydrogel for emergency hemostasis and long‐term management of trauma wounds. (a) ACmCS self‐assembles into particulate structures via electrostatic interactions in deionized water. (b) The synthesis of PACmC hydrogel and its mechanism for rapid hemostasis and long‐term stable sealing. (c) PACmC hydrogel achieves rapid, robust and long‐term adhesion in porcine models of hepatic and splenic hemorrhage.

Herein, we have developed an injectable hydrogel (denoted as PACmC) with rapid coagulation, ultrahigh burst pressure tolerance, and low swelling properties for emergency hemostasis and long‐term management of trauma wounds by the aminated carboxymethyl chitosan (ACmCS) nanoparticles and their crosslinking with N‐hydroxysuccinimide (NHS) ester‐terminated four‐arm polyethylene glycol (Tetra‐PEG‐SS) through efficient NHS‐amine coupling (Figure 1). ACmCS exhibits excellent water solubility and effectively restores the active coagulation capability of chitosan mediated by charge interactions. Furthermore, amino grafting in an appropriate proportion enables ACmCS to self‐assemble in deionized water, forming stable nanoparticles that distribute as “rigid island” within the hydrogel network, which effectively inhibits water penetration and network expansion at the physical level. On the other hand, as the “flexible chain”, Tetra‐PEG‐SS can simultaneously achieve the in‐situ crosslinking of the ACmCS nanoparticles and adhesive anchoring to the tissue surface, thereby forming a stable physical hemostatic barrier. This unique “rigid island‐flexible chain” structure endows PACmC hydrogel with a high mechanical performance (an elastic modulus of 211.9 kPa) and outstanding anti‐swelling capability (a swelling ratio of only 49.2% within 7 d). The efficient crosslinking reaction drives rapid in‐situ network formation (with a gelation time of 22.3 s at room temperature) and establishes strong interfacial adhesion (an adhesion strength of 46.5 kPa) along with ultrahigh burst pressure tolerance (up to 701 mm Hg) on irregular wound surfaces, laying the foundation for rapid hemostasis and long‐term sealing. The efficacy of our PACmC hydrogel is evaluated in New Zealand rabbit models of liver and spleen hemorrhage, demonstrating marked superiority over commercial hemostats (thrombin powder and gauze) in achieving rapid hemostasis and effective sealing. To further evaluate its clinical potential, preclinical studies in porcine models of severe hepatic and splenic trauma are performed. These results consistently validate PACmC hydrogel's outstanding hemostatic capability and sustained sealing performance. This study provides a clear technological pathway from molecular functional design and controlled nano‐assembly to macroscopic structural construction, offering an innovative research paradigm for the development of advanced injectable hemostatic materials.

## 2 Results and Discussion

### 2.1 Preparation and Physical Characterization of PACmC Hydrogel

Chitosan‐based hemostatic materials have emerged as primary agents for emergency bleeding control, owing to their good biocompatibility, degradability and rapid coagulation mechanism based on polycationic properties. However, their inherent poor solubility, relatively high brittleness, and inadequate anti‐swelling capacity often limit the development of injectable formulations. Common modified derivatives with polyampholyte characteristics, such as carboxymethyl chitosan (CmCS), have improved solubility and anti‐swelling performance. Nevertheless, the introduction of anionic groups typically leads to the loss of chitosan's intrinsic cationic procoagulant function. To address this “functional exclusivity” issue, ACmCS is prepared via controlled amination of CmCS, which retains excellent water solubility and restores a strongly positive surface charge for the active coagulation capability. Interestingly, ACmCS can further self‐assemble in deionized water to form nanoparticles with uniform size and stable structure. Subsequently, through the rapid click chemical reaction between the NHS ester groups of Tetra‐PEG‐SS and amino groups of ACmCS and tissue, in‐situ gelation and adhesion anchoring on tissue surfaces can be simultaneously achieved, forming a PACmC hydrogel barrier for hemostatic. In this design, the “rigid island” contributes high mechanical strength and anti‐swelling capacity, while the “flexible chains” provide adhesion and toughness. Their synergy reconciles at the molecular level the performance contradictions that were previously difficult to balance.

**FIGURE 2:**
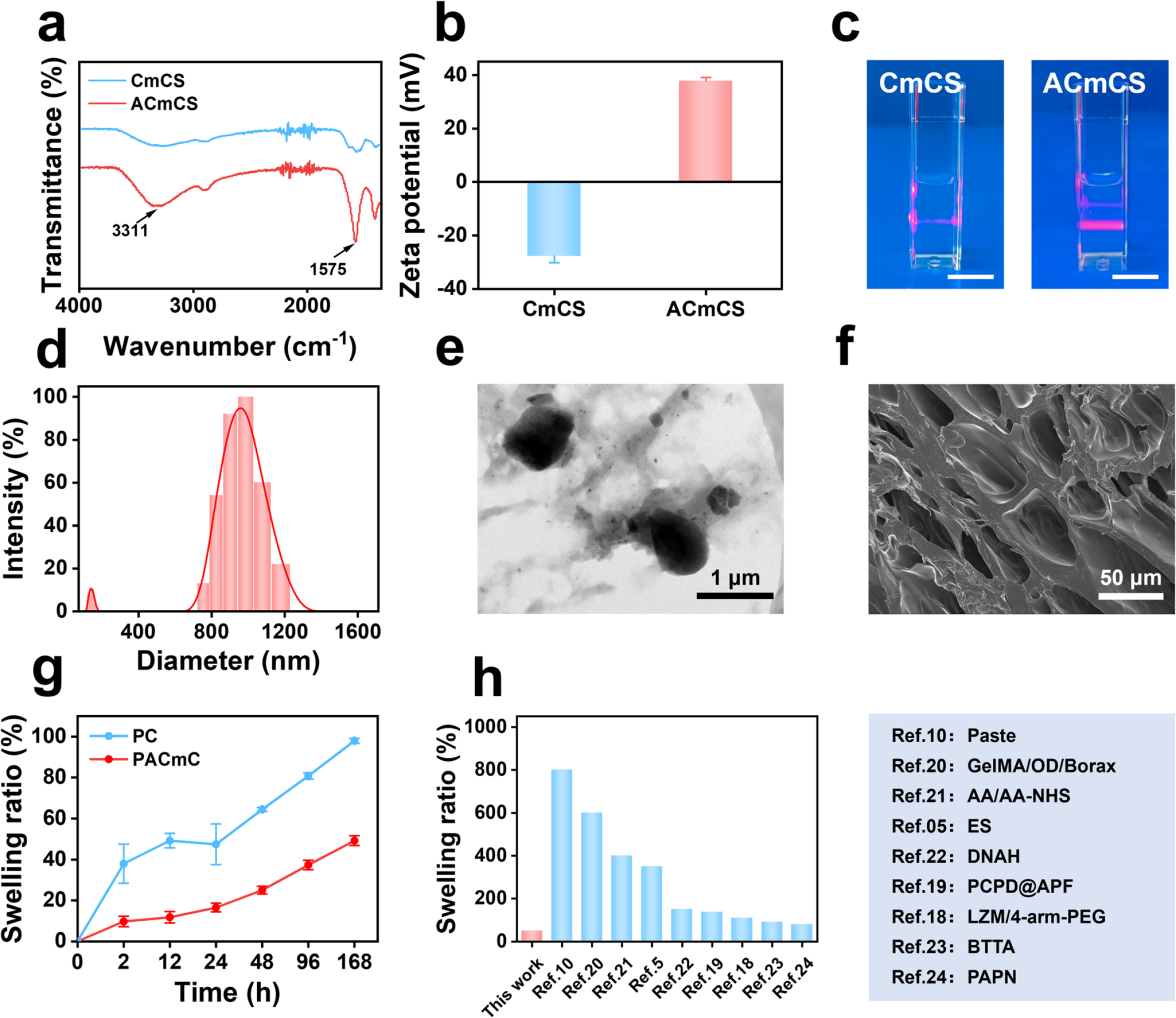
Physical characterizations of PACmC hydrogel. (a) FTIR spectra of CmCS and ACmCS. (b) Zeta potential of CmCS and ACmCS. The data are the mean ± standard deviation (SD, *n* = 3 independent samples). (c) Mie scattering of CmCS and ACmCS. Scale bars: 10 mm. (d) Particle size distribution of ACmCS. (e) TEM image of ACmCS nanoparticle. (f) SEM image of the cross‐section of lyophilized PACmC hydrogel. (g) Swelling ratios of PC and PACmC hydrogels in PBS at 37°C for 7 d. The data are the mean ± SD (*n* = 3 independent samples). (h) Swelling ratios of PACmC hydrogel in PBS for 7 d and some recently reported wet adhesives.

To confirm the successful preparation of ACmCS, Fourier‐transform infrared (FTIR) spectroscopy was performed. Compared with CmCS, the spectrum of ACmCS exhibits significant enhancement of the peaks at 3311 and 1575 cm^−^^1^ (Figure 2a). The broad peak at 3311 cm^−^^1^ is attributed to overlapping O‐H and N‐H stretching vibrations, while the strong peak at 1575 cm^−^^1^ corresponds to the amide II band (Figure 2a). The Zeta potential of ACmCS shifts significantly to 37.9 ± 1.2 mV compared to unmodified CmCS (−27.7 ± 2.5 mV), also confirming the introduction of amine groups and thus the successful preparation of ACmCS (Figure 2b). In contrast to CmCS, ACmCS exhibits a pronounced Mie scattering under the equivalent concentration (0.1 mg mL^−1^, Figure 2c), indicating the presence of colloidal particles. We further analyzed the aggregation mechanism of ACmCS. As shown in Figure S1, Mie scattering of ACmCS is significantly diminished upon pH adjustment, while adding urea or Tween 80 causes no obvious change. This result indicates that the self‐assembly of ACmCS is primarily driven by electrostatic interactions rather than hydrophobic effects or hydrogen bonding. Particle size analysis shows that ACmCS particles are predominantly distributed between 800−1000 nm (Figure 2d). Notably, even at a 100‐fold higher concentration, the self‐assembled ACmCS nanoparticles remained predominantly distributed within the similar size range, demonstrating excellent aqueous stability (Figure S2). Furthermore, transmission electron microscopy (TEM) images reveal uniform ACmCS particles, corroborating its ability to form self‐assembled nanoscale colloidal structures in deionized water (Figure 2e).

PACmC hydrogel was prepared by mixing equal volumes of Tetra‐PEG‐SS solution and ACmCS colloidal dispersion. With a fixed Tetra‐PEG‐SS mass fraction, increasing the ACmCS mass fraction can shorten the gelation time, which is attributed to the increased number of available crosslinking sites. Through the mixing of 7 wt% ACmCS colloidal dispersion and 15 wt% Tetra‐PEG‐SS solution, a gelation time of 22.3 ± 2.1 s is achieved for PACmC hydrogel, significantly shorter than the gelation time of PC hydrogel (replaced ACmCS with CmCS, approximately 70.0 ± 4.6 s) under the same conditions (Figure S3). Through optimization of the mass ratio, PACmC hydrogel is endowed with rapid gelation capability. Furthermore, PACmC hydrogel exhibits excellent moldability and injectability, allowing it to be easily shaped into various forms using molds and to form specific letter shapes through continuous injection (Figure S4). Time‐sweep analysis further confirms that PACmC hydrogel formed under these optimized conditions displays a gelation time of approximately 20 s, which is highly consistent with the results obtained by the tube inversion method. In addition, the hydrogel demonstrates stable solid‐like gel behavior in frequency‐sweep analysis (Figure S5). These properties collectively ensure its potential for precise filling and stable maintenance in complex defect sites. Scanning electron microscopy (SEM) images reveal that lyophilized PACmC hydrogel possesses a regularly porous structure with uniform pore distribution (Figure 2f). This interconnected porous network could provide a conducive scaffold for subsequent cell adhesion and proliferation after hemostasis [17].

Since low swelling is crucial for achieving long‐term adhesion in wet environments, the swelling behavior was evaluated [18, 19]. In contrast to PC hydrogel (98.0% ± 1.3%), PACmC hydrogel exhibits a good anti‐swelling performance, with a swelling ratio as low as 49.2% ± 2.4% within 7 d (Figure 2g; Figure S6). To the best of our knowledge, the swelling ratio of PACmC hydrogel is significantly lower than those of currently reported wet‐adhesive hydrogels (Figure 2h) [5, 10, 18, 19, 20, 21, 22, 23, 24]. This improvement is attributed to the “rigid island‐flexible chain” structure formed by the electrostatically self‐assembled ACmCS nanoparticles and the flexible PEG chains, which effectively restricts water infiltration and chain extension, thereby ensuring structural and functional stability in physiological environments [25, 26]. Taken together, the amination of carboxymethyl chitosan gives rise to ACmCS capable of electrostatic self‐assembly into a nanoparticle‐reinforced network, thereby imparting the hydrogel with distinctive anti‐swelling behavior.

### 2.2 Interfacial Adhesion and Intrinsic Mechanical Properties of PACmC Hydrogel

**FIGURE 3:**
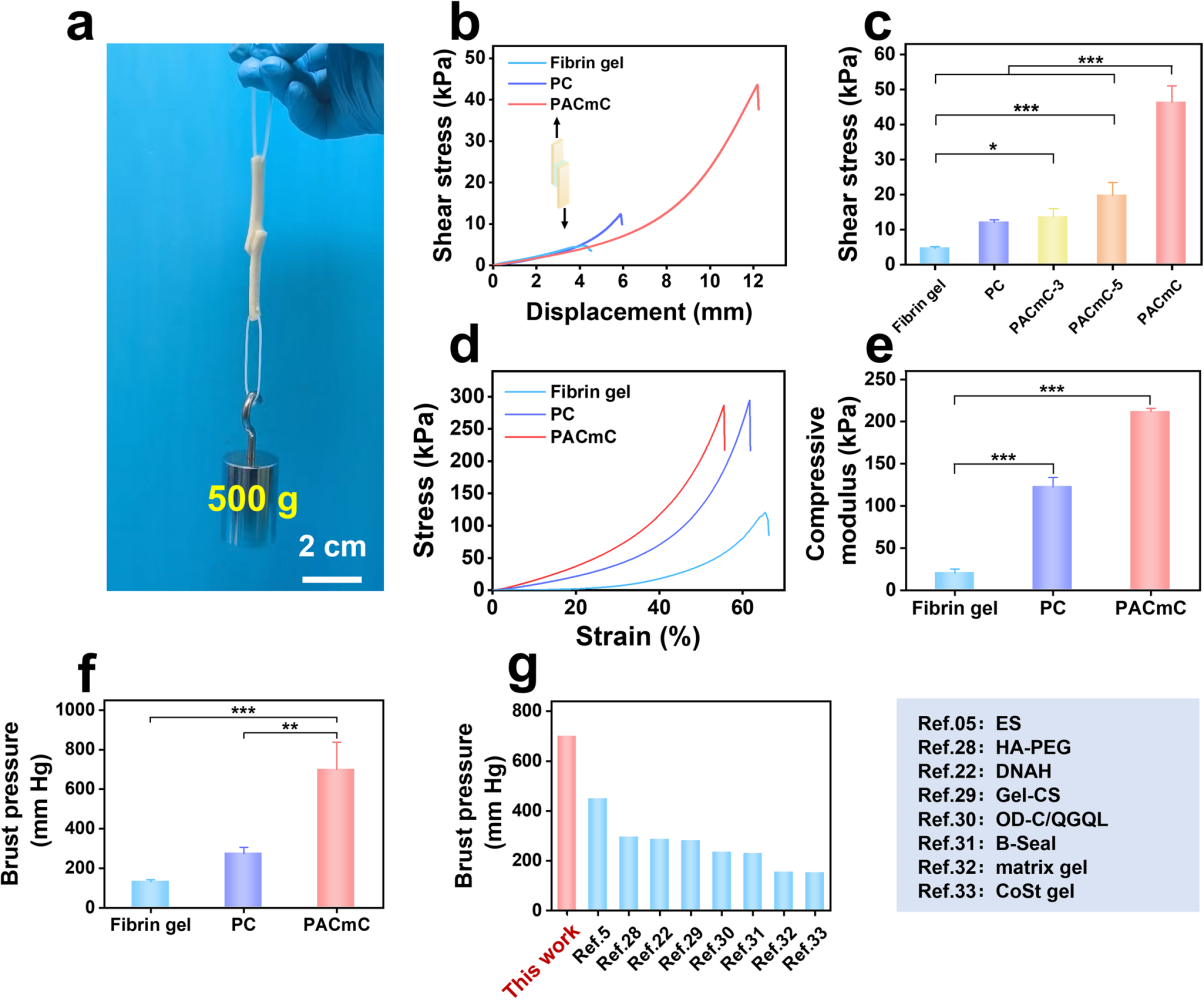
Interfacial adhesion and intrinsic mechanical properties of PACmC hydrogel. (a) Digital photos of weight‐lifting test of PACmC hydrogel. (b,c) Shear stress–displacement curves (b) and shear stress (c) for lap‐shear test of porcine skin adhered by Fibrin gel, PC (prepared from 7 wt% CmCS solution), PACmC‐3 (3 wt% ACmCS), PACmC‐5 (5 wt% ACmCS), and PACmC (7 wt% ACmCS) hydrogels. The data are the mean ± SD (*n* = 3 independent samples; data are analyzed using one‐way ANOVA; * *p* < 0.05, *** *p* < 0.001). (d,e) Compressive stress‐strain curves (d) and compressive modulus (e) of Fibrin gel, PC hydrogel, and PACmC hydrogel. The data are the mean ± SD (*n* = 3 independent samples; data are analyzed using one‐way ANOVA; *** *p* < 0.001). (f) Burst pressures of Fibrin gel, PC hydrogel, and PACmC hydrogel on dissected porcine skin. The data are the mean ± SD (*n* = 3 independent samples; data are analyzed using one‐way ANOVA; ** *p* < 0.01, *** *p* < 0.001). (g) Burst pressures of PACmC hydrogel and some recently reported wet adhesives.

To evaluate the adhesion performance of PACmC hydrogel, we employed a series of macroscopic observations and various mechanical tests. The results show that the PACmC hydrogel forms a firm bridge connecting the hepatic and splenic tissues to the glove, and remains intact without peeling or detaching (Figure S7a). In addition, PACmC hydrogel adheres tightly to porcine skin, withstanding bending, stretching, twisting, and rinsing (Figure S7b). Its macroscopic adhesive strength was further assessed via a weight‐lifting test. A 1 cm × 1 cm PACmC hydrogel was sandwiched between two pieces of fresh porcine skin. The bonded sample successfully lifts a 500 g weight, confirming its superior adhesive performance (Figure 3a).

To quantify the adhesive capability, lap‐shear tests were conducted. PACmC hydrogel with the mixing of 7 wt% ACmCS colloidal dispersion and 15 wt% Tetra‐PEG‐SS solution exhibits the highest lap‐shear strength of 46.5 ± 4.6 kPa, which is significantly higher than that of both PC hydrogel (12.0 ± 0.7 kPa) and commercial Fibrin gel (4.6 ± 0.5 kPa), representing an approximately ten‐fold enhancement over Fibrin gel (Figure 3b,c). The intrinsic mechanical properties were evaluated by uniaxial compression tests. PACmC hydrogel can withstand pressures up to 286.8 kPa, with an elastic modulus of 211.9 ± 3.8 kPa within the 10%–20% strain range (Figure 3d,e). This modulus is higher than that of PC hydrogel (122.5 ± 11.1 kPa, Figure 3d,e), which may be attributed to the “rigid island” structure formed by the self‐assembled ACmCS nanoparticles, providing abundant rigid reinforcing domains within the covalently crosslinked hydrogel network and enhancing the compressive modulus through nanocomposite reinforcement. The modulus of PACmC hydrogel matches the mechanical properties of human liver tissues (196.5 ± 13.2 kPa), indicating excellent structural compliance suitable for dynamic physiological conditions [27].

To evaluate the hydrogel's pressure resistance under simulated physiological conditions, a burst pressure test was conducted. As shown in Figure S8, PACmC hydrogel was applied to seal a 3 mm diameter through‐hole in porcine skin. After gelling for 10 min, the sealed sample was mounted in a fixture, and deionized water was infused at 1 mL min^−^^1^. The seal remains intact until the burst pressure reaches 701 ± 137 mm Hg (Figure 3f; Video S1), significantly exceeding that of PC (275 ± 32 mm Hg) and commercial Fibrin gel (132 ± 10 mm Hg, Figure 3f). This exceptional performance may be attributed to the “rigid island‐flexible chain” architecture formed by the self‐assembled ACmCS nanoparticles, which enhances the hydrogel's resistance against pressure‐induced failure. It is worth mentioning that such a high burst pressure is much superior to those of recently reported injectable wet‐adhesive hydrogels (Figure 3g) [5, 22, 28, 29, 30, 31, 32, 33].

**FIGURE 4:**
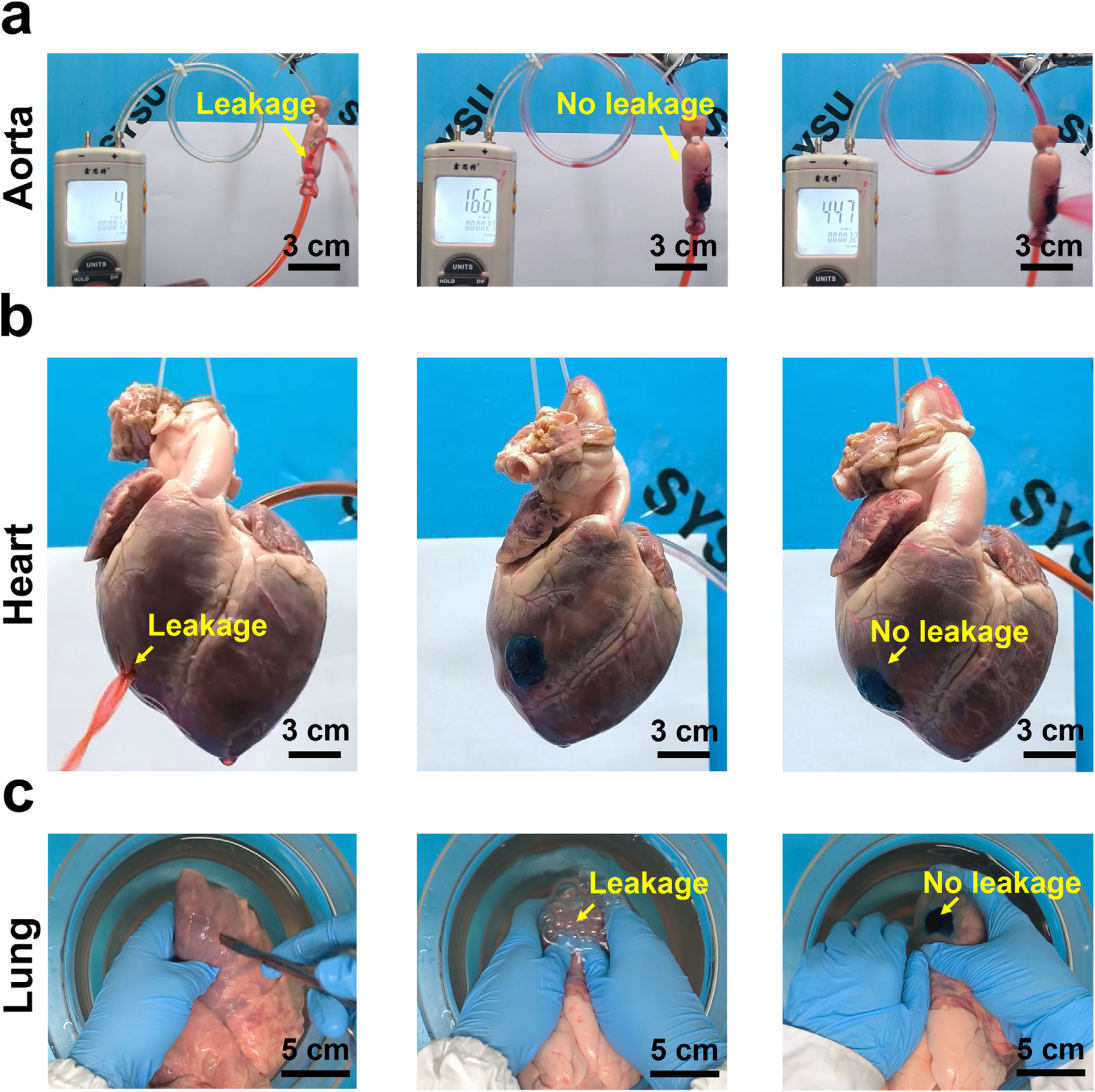
The sealing capability of PACmC hydrogel on injured and leaking organs. (a,b) Liquid sealing performances of PACmC hydrogel on perforated porcine aorta (a) and ruptured porcine heart (b). (c) Air sealing performance of PACmC hydrogel on dissected porcine lung.

As a preliminary assessment of tissue adhesion, we evaluated the sealing capability of PACmC hydrogel using ex vivo tissue models under simulated conditions, prior to further validation in dynamic in vivo bleeding models. To evaluate the sealing performance, various ex vivo tissue perforation models were established. In liquid sealing tests, PACmC hydrogel rapidly forms a robust adhesion to wet tissues, completely preventing liquid leakage from perforated porcine aortas and fluid‐filled ruptured porcine hearts under high pressure (Figure 4a,b). Notably, in the porcine aorta perforation model, a burst pressure test shows that PACmC hydrogel can withstand pressures up to 447 mm Hg, remarkably exceeding the normal human systolic arterial pressure range (90–140 mm Hg, Video S2) [34]. For gas sealing, PACmC hydrogel also exhibits excellent airtightness on ex vivo lung tissue (Figure 4c). These results demonstrate the outstanding adhesive sealing capability of PACmC hydrogel.

### 2.3 Coagulation Ability, Biocompatibility, and Biodegradation of PACmC Hydrogel

**FIGURE 5:**
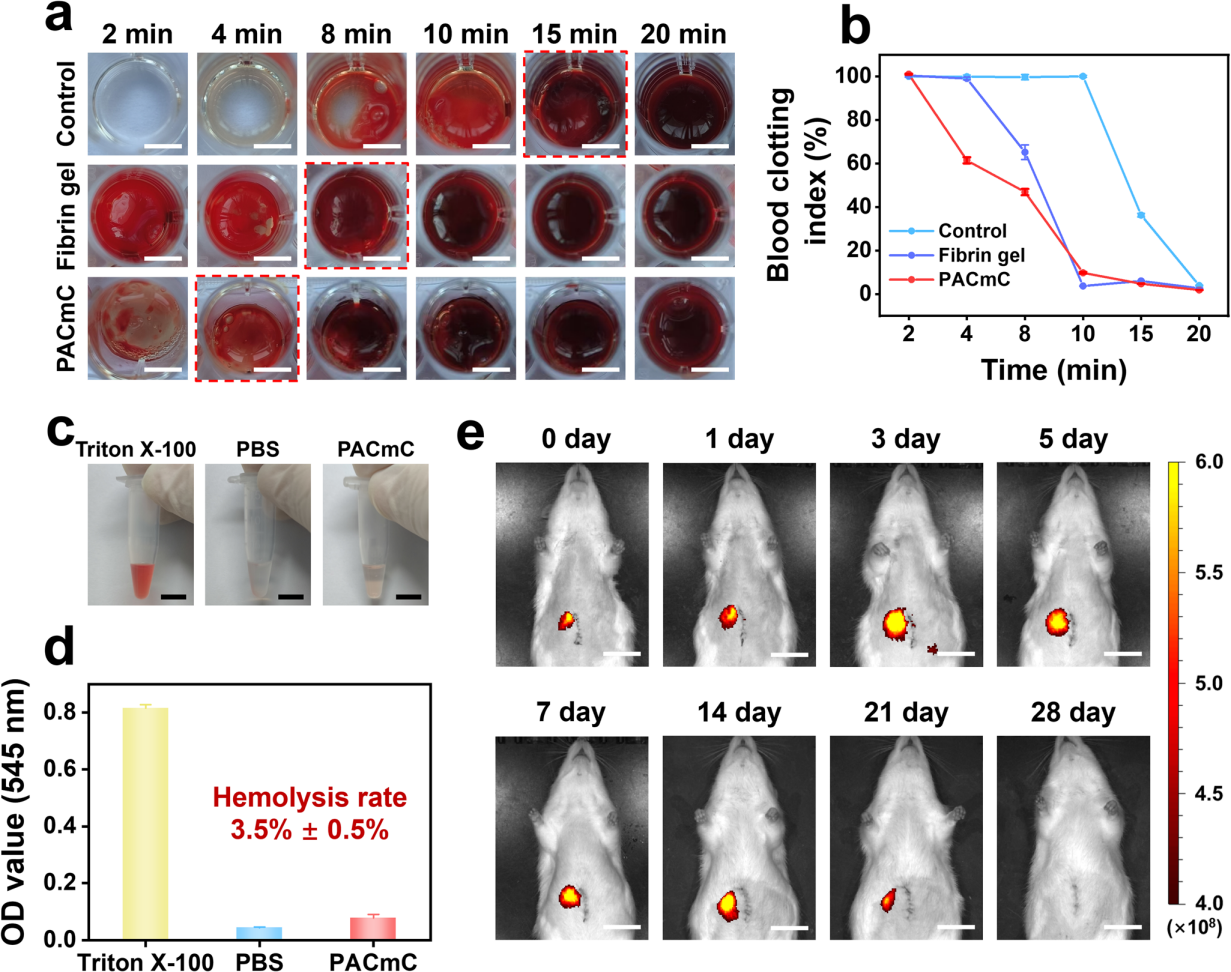
Clotting ability, biocompatibility, and biodegradation of PACmC hydrogel. (a) Time‐dependent clotting of uncoated (control), Fibrin gel‐coated, and PACmC hydrogel‐coated wells. Scale bars: 5 mm. (b) Blood clotting index of each group as a function of incubation time. The data are the mean ± SD (*n* = 3 independent samples). (c) Digital photos of the hemolysis of erythrocytes co‐incubation with Triton X‐100, PBS, and PACmC hydrogel for 4 h. Scale bars: 10 mm. (d) Hemolysis rate of PACmC hydrogel. The data are the mean ± SD (*n* = 3 independent samples). (e) Representative images showing the time‐dependent degradation of PACmC hydrogel labeled by Cy5.5 NHS ester on the liver of rats in vivo. Scale bars: 3 cm.

An effective hemostatic material should rapidly induce blood coagulation and achieve minimum damage to blood cells [35, 36]. To evaluate the coagulation ability of PACmC hydrogel, coagulation time and the optical density (OD) of the supernatant post‐coagulation were measured by coating 48‐well plates with the hydrogel and adding whole blood. In wells coated with PACmC hydrogel, blood coagulation time initiates within 4 min, much shorter than that of the commercial Fibrin gel and uncoated control groups (8 min and 15 min, respectively, Figure 5a,b). Additionally, we observed PACmC hydrogel samples after contact with blood using cryo‐scanning electron microscopy. The results showed extensive aggregation of red blood cells and platelets on the hydrogel surface, further demonstrating its excellent coagulation ability (Figure S9). The rapid coagulation capability of PACmC hydrogel is attributed to the cationic nature of ACmCS, which enables effective electrostatic adsorption of erythrocytes and platelets [37].

Good biocompatibility is a prerequisite for the in vivo application of hemostatic material. CCK‐8 cell viability assays and live/dead cell fluorescence staining show that the survival rate of L929 cells co‐cultured with PACmC hydrogel for 3 d exceeded 92.7%, showing no significant difference from the control group (Figure S10a,b). The excellent cytocompatibility of PACmC hydrogel is attributed to the inherent biocompatibility of chitosan and PEG derivatives. To further assess in vivo biocompatibility, PACmC hydrogel is subcutaneously implanted into the dorsal region of rats. PACmC hydrogel elicits a mild inflammatory response with no obvious cytotoxicity, which has no significant difference compared to the control group (Figure S10c,d). Furthermore, hemocompatibility analysis was performed. As shown in Figure 5c,d, the hemolysis rate after incubating PACmC hydrogel with red blood cell supernatant for 4 h is approximately 3.5%, which is below the 5% safety threshold stipulated by international standards for medical materials, indicating ideal hemocompatibility of PACmC hydrogel [36].

To evaluate the in vivo degradation behavior, PACmC hydrogel was fluorescently labeled with Cy5.5 NHS ester and implanted into the liver site of rats. The fluorescence signal intensity and distribution gradually decrease over 28 d post‐implantation, and eventually fall below the detectable threshold for meaningful fluorescence (Figure 5e). Notably, the same amide bond chemistry is used for both Cy5.5 labeling and hydrogel crosslinking. Therefore, this fluorescence decay trend suggests gradual degradation of the hydrogel network in vivo, indicating that PACmC hydrogel not only achieves long‐term stable adhesion on the liver surface but also gradually resorbs over time. These results collectively demonstrate the in vivo application potential and safety of PACmC hydrogel as a hemostatic material.

### 2.4 Hemostasis and Sealing of Massive Hemorrhage in Rabbits

**FIGURE 6:**
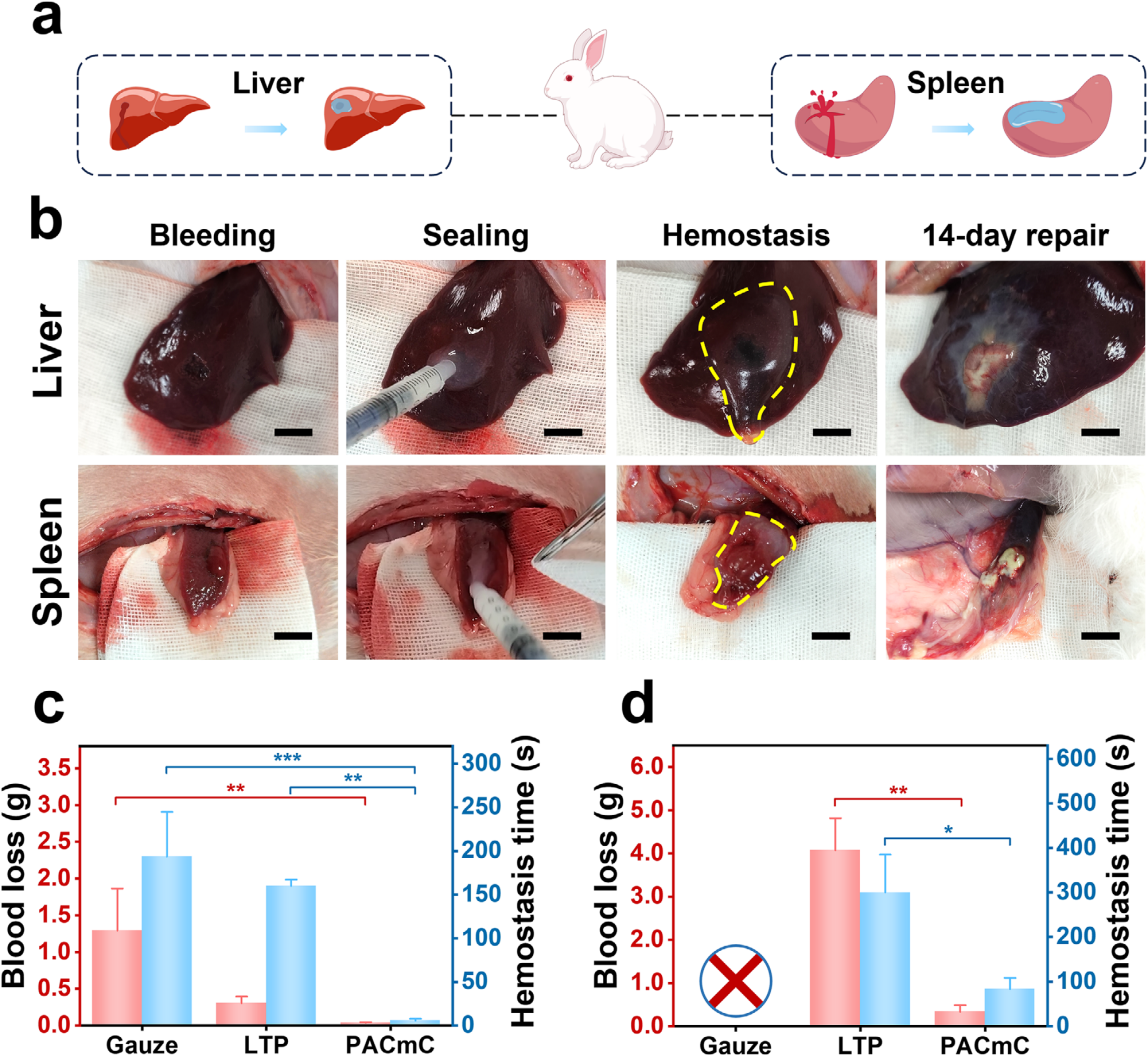
Hemostatic efficacy of PACmC hydrogel in hepatic and splenic injury bleeding models of New Zealand rabbits. (a) Illustration of hepatic volumetric defect and splenic incision injury hemorrhage models. (b) Digital photos of bleeding, hemostatic and 14 d wound healing process of rabbit liver and spleen by PACmC hydrogel, respectively. Scale bars: 10 mm. (c,d) Comparison of total blood loss and hemostatic time in hepatic (c) and splenic (d) injury models of rabbits treated using gauze, lyophilized thrombin powder, and PACmC hydrogel (red crosses indicate the failure to stop bleeding). The data are the mean ± SD (*n* = 3 independent samples; data are analyzed using one‐way ANOVA; * *p* < 0.05, ** *p* < 0.01, *** *p* < 0.001).

To validate the in vivo hemostatic and sealing capability of PACmC hydrogel, hepatic and splenic hemorrhage models were established in New Zealand rabbits (Figure 6a). Active bleeding is induced by surgically creating a circular liver defect (10 mm diameter × 5 mm depth) and a spleen linear laceration (10 mm length × 5 mm depth, Figure 6b). The hemostatic efficacy of the PACmC hydrogel group is evaluated against the control groups treated with clinically common lyophilized thrombin powder (LTP) or gauze compression by measuring blood loss and hemostasis time [38]. In the liver hemorrhage model, the gauze and LTP groups show limited hemostatic efficacy, with hemostasis times of approximately 193.7 and 160.0 s, and blood losses of 1.3 and 0.3 g, respectively (Figure 6c). Notably, LTP forms only a superficial clot that fails to halt underlying active bleeding (Video S3). This incomplete hemostasis meant that physiological movement could dislodge the fragile clot during organ repositioning, posing a re‐bleeding risk. In sharp contrast, pre‐mixed PACmC hydrogel precursors gel in situ within approximately 5.7 s upon injection, providing an effective hemostatic seal with a blood loss of only about 0.04 g (Figure 6c; Video S4), demonstrating markedly superior performance.

To further examine the excellent hemostatic efficacy of PACmC hydrogel in more fragile and highly vascularized splenic tissues, a splenic injury and hemorrhage model was established. Gauze compression fails to control bleeding within 7 mins (Figure 6d). In the LTP group, 50% of subjects fail to achieve effective hemostasis, as the weak clot barrier ruptures under bleeding pressure (Video S5). Even in successful cases, LTP is prone to displacement by sustained hemorrhage, necessitating repeated application and resulting in prolonged hemostasis time (300.0 s) and high blood loss (4.1 g, Figure 6d). Encouragingly, PACmC hydrogel achieves rapid hemostasis in the splenic hemorrhage model within approximately 83.7 s, with minimal blood loss (0.3 g), and effectively seals the laceration without subsequent oozing (Figure 6b,d; Video S6).

To evaluate tissue repair following PACmC hydrogel treatment, hepatic and splenic samples were harvested 14 d post‐operation. No significant adhesions are formed between the treated tissues and surrounding abdominal organs, and most of the hydrogel initially covering the wounds have degraded (Figure 6b). Hematoxylin and eosin (HE) and Masson staining show intact hepatic lobules and splenic sinuses with normal architecture and significant collagen deposition at the material‐tissue interface, indicating good tissue healing (Figure S11). These observations align with the favorable biocompatibility and degradability of PACmC hydrogel. This positive outcome is likely attributable to the porous scaffold structure and degradation behavior of PACmC hydrogel, which together provide a conducive microenvironment for cell adhesion and proliferation [39, 40, 41]. These results demonstrate that PACmC hydrogel not only possesses excellent hemostatic sealing ability, but also achieves long‐term, stable sealing and repair in vivo, presenting an ideal therapeutic strategy for hemostasis.

### 2.5 Hemostasis and Sealing of Massive Hemorrhage in a Pig

**FIGURE 7:**
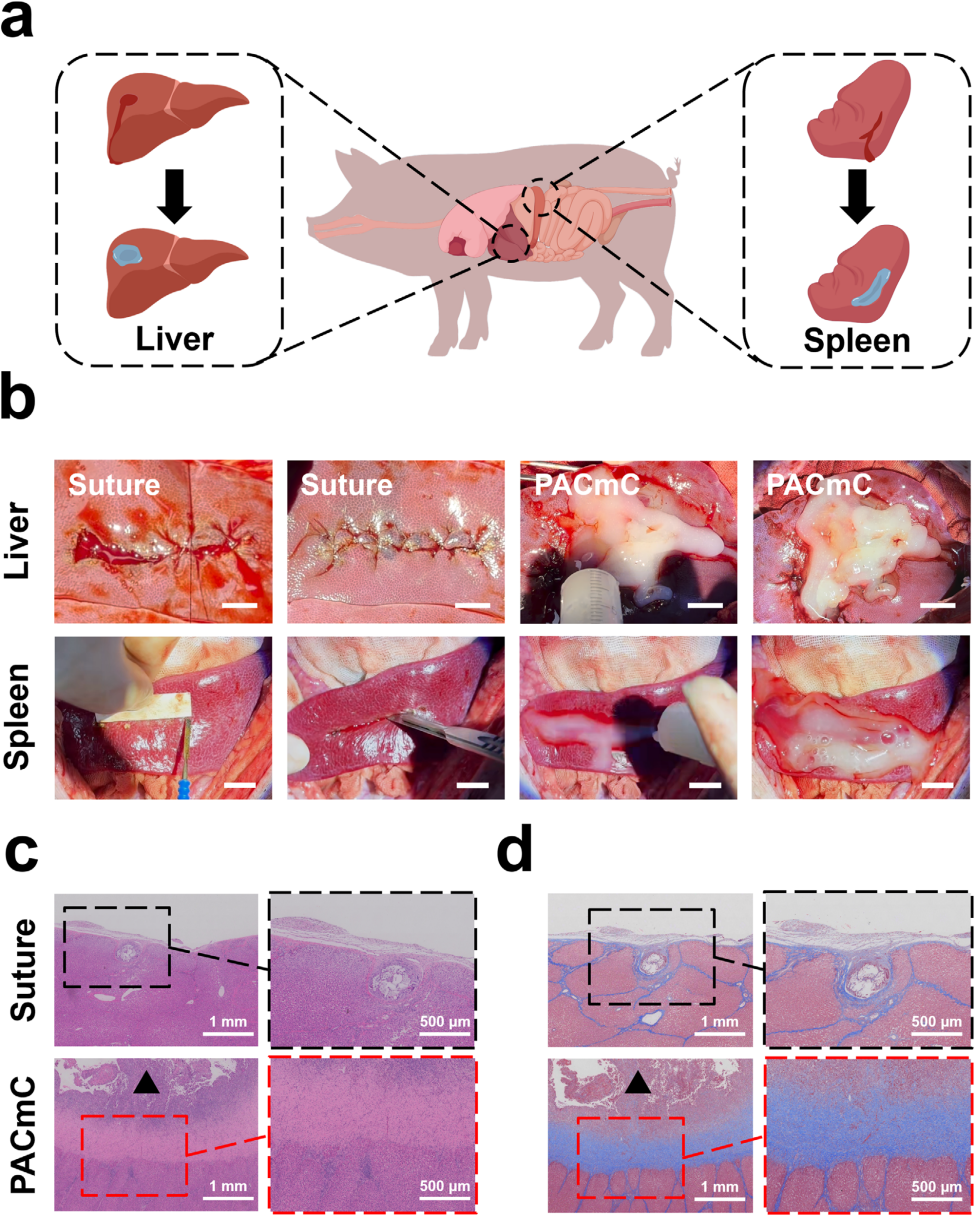
Hemostatic efficacy of PACmC hydrogel in hepatic and splenic injury bleeding models of a pig. (a) Illustration of the hepatic volumetric defect, and splenic incision injury hemorrhage models. (b) Digital photos of the treatment for porcine hepatic and splenic injury hemorrhage models. Scale bars: 10 mm. (c,d) HE (c) and Masson (d) staining images of the injured liver tissues after the treatment of suture and PACmC hydrogel at 14 d. Triangular markings indicate the approximate locations of PACmC hydrogel occlusion on the liver surface.

To better approximate clinical settings, we established porcine models of hepatic defect and splenic traumatic hemorrhage to validate the hemostatic sealing and long‐term repair performance of PACmC hydrogel in severe trauma (Figure 7a). The hemostasis time of the PACmC hydrogel group on hepatic defect bleeding is significantly shortened to 147 s, while that of the suture group is 346 s (Figure 7b). Concurrently, the blood loss of the PACmC hydrogel group (7.1 g) is significantly lower than that of the suture group (23.0 g, Figure S12). To further examine the excellent hemostatic efficacy of PACmC hydrogel in more fragile and highly vascularized splenic tissues, a major laceration (30 mm length × 5 mm depth) was created to simulate substantial active bleeding (Figure 7a,b). Consistently, PACmC hydrogel can still achieve effective hemostatic sealing within approximately 90 s, verifying its reliable sealing capability on wet tissue surfaces (Figure 7b; Video S7). The pigs recover normally post‐surgery and survive in good health throughout the 14‐day observation period. To investigate the long‐term repair performance of PACmC hydrogel, tissue samples from treated liver and spleen were collected at 14 d for histopathological analysis. Compared to the suture group, HE and Masson staining indicate that the PACmC hydrogel group exhibits the favorable healing on hepatic injury surface with PACmC hydrogel on the liver surface having largely degraded in vivo (Figure 7c,d). In addition, the internal structure of the liver remained intact, and the morphology of the hepatic lobules was normal (Figure S13). Similarly, the injury splenic tissue achieves a good healing with only minimal collagen deposition and clear splenic sinus structure (Figure S14). These results demonstrate that PACmC hydrogel can establish a robust and long‐term seal on hepatic and splenic massive hemorrhagic wounds in the wet and dynamic environments.

## 3 Conclusion

In summary, we have developed an injectable PACmC hydrogel that integrates rapid coagulation, ultrahigh burst pressure tolerance, and low swelling for emergency hemostasis and long‐term wound management. To achieve this integration, we have designed cationic nanoparticles via electrostatic self‐assembly of ACmCS, which restore procoagulant activity while maintaining excellent solubility. These nanoparticles are then rapidly crosslinked with Tetra‐PEG‐SS through NHS‐amine coupling, yielding in situ gelation within 22.3 s. The densely packed ACmCS nanoparticle network physically restricts water infiltration, resulting in an outstanding low swelling ratio of only 49.2% within 7 d. Meanwhile, the NHS‐ester groups on Tetra‐PEG‐SS covalently bond with amino groups of the tissue, and the ACmCS nanoparticles serve as multivalent crosslinking centers that enhance cohesive strength, together enabling robust wet‐tissue adhesion (46.5 kPa) and an ultrahigh burst pressure tolerance of 701 mm Hg. The cationic ACmCS nanoparticles further accelerate hemostasis by actively aggregating blood components. In rabbit and porcine models of hepatic and splenic hemorrhage, our PACmC hydrogel significantly reduces blood loss and hemostasis time compared to clinical hemostats (thrombin powder and gauze), while maintaining long‐term sealing with good biocompatibility and gradual degradation. Our work could offer a new conceptual and practical framework for developing high‐performance injectable hemostatic materials applicable to emergency hemorrhage control and soft tissue wound repair.

## 4 Experimental Section

The experimental section is available in the Supporting Information.

## Conflicts of Interest

The authors declare no conflicts of interest.

## Supporting information




**Supporting File 1**: advs75329‐sup‐0001‐SuppMat.docx.

**Supporting File 2**: advs75329‐sup‐0002‐MovieS1.mp4.

**Supporting File 3**: advs75329‐sup‐0003‐MovieS2.mp4.

**Supporting File 4**: advs75329‐sup‐0004‐MovieS3.mp4.

**Supporting File 5**: advs75329‐sup‐0005‐MovieS4.mp4.

**Supporting File 6**: advs75329‐sup‐0006‐MovieS5.mp4.

**Supporting File 7**: advs75329‐sup‐0007‐MovieS6.mp4.

**Supporting File 8**: advs75329‐sup‐0008‐MovieS7.mp4.

## Data Availability

The data that support the findings of this study are available from the corresponding author upon reasonable request.

## References

[1] S. J. Stanworth , R. Davenport , N. Curry , et al., “Mortality From Trauma Haemorrhage and Opportunities for Improvement in Transfusion Practice,” British Journal of Surgery 103 (2016): 357–365, 10.1002/bjs.10052. 26841720

[2] J. W. Cannon , “Hemorrhagic Shock,” New England Journal of Medicine 378 (2018): 370–379, 10.1056/NEJMra1705649. 29365303

[3] G. M. Taboada , K. Yang , M. J. N. Pereira , et al., “Overcoming the Translational Barriers of Tissue Adhesives,” Nature Reviews Materials 5 (2020): 310–329, 10.1038/s41578-019-0171-7.

[4] B. Guo , R. Dong , Y. Liang , and M. Li , “Haemostatic Materials for Wound Healing Applications,” Nature Reviews Chemistry 5 (2021): 773–791, 10.1038/s41570-021-00323-z. 37117664

[5] Y. Yang , G. He , Z. Pan , et al., “An Injectable Hydrogel With Ultrahigh Burst Pressure and Innate Antibacterial Activity for Emergency Hemostasis and Wound Repair,” Advanced Materials 36 (2024): 2404811, 10.1002/adma.202404811. 38875445

[6] S. Cao , K. Zhang , Q. Li , S. Zhang , and J. Chen , “Injectable and Photothermal Antibacterial Bacterial Cellulose Cryogel for Rapid Hemostasis and Repair of Irregular and Deep Skin Wounds,” Carbohydrate Polymers 320 (2023): 121239, 10.1016/j.carbpol.2023.121239. 37659822

[7] A. Sanyal , S. Roy , A. Ghosh , M. Chakraborty , A. Ghosh , and D. Mandal , “The Next Frontier in Hemorrhagic Management: A Comprehensive Review on Development of Natural Polymer‐Based Injectable Hydrogels as Promising Hemostatic Dressings,” Chemical Engineering Journal 497 (2024): 155033, 10.1016/j.cej.2024.155033.

[8] X. Yang , X. Wang , L. Tang , et al., “Water Triggered Injectable Polylactic Acid Hydrogel Based on Zwitterionic Sulfobetaine Modification for Incompressible Bleeding and Tissue Anti‐Adhesion,” Materials Today Bio 30 (2024): 101431, 10.1016/j.mtbio.2024.101431. PMC11742595 39830134

[9] C. Cui , C. Fan , Y. Wu , et al., “Water‐Triggered Hyperbranched Polymer Universal Adhesives: From Strong Underwater Adhesion to Rapid Sealing Hemostasis,” Advanced Materials 31 (2019): 1905761, 10.1002/adma.201905761. 31625635

[10] H. Yuk , J. Wu , T. L. Sarrafian , et al., “Rapid and Coagulation‐Independent Haemostatic Sealing by a Paste Inspired by Barnacle Glue,” Nature Biomedical Engineering 5 (2021): 1131–1142, 10.1038/s41551-021-00769-y. PMC9254891 34373600

[11] C. Fu , L. Shen , L. Liu , et al., “Hydrogel With Robust Adhesion in Various Liquid Environments by Electrostatic‐Induced Hydrophilic and Hydrophobic Polymer Chains Migration and Rearrangement,” Advanced Materials 35 (2023): 2211237, 10.1002/adma.202211237. 36662770

[12] V. S. Raghuwanshi and G. Garnier , “Characterisation of Hydrogels: Linking the Nano to the Microscale,” Advances in Colloid and Interface Science 274 (2019): 102044, 10.1016/j.cis.2019.102044. 31677493

[13] E. Filippidi , T. R. Cristiani , C. D. Eisenbach , et al., “Toughening Elastomers Using Mussel‐Inspired Iron‐Catechol Complexes,” Science 358 (2017): 502–505, 10.1126/science.aao0350. 29074770 PMC5676464

[14] Y. Liu , G. Guan , Y. Li , et al., “Gelation of Highly Entangled Hydrophobic Macromolecular Fluid for Ultrastrong Underwater In Situ Fast Tissue Adhesion,” Science Advances 8 (2022): abm9744, 10.1126/sciadv.abm9744. PMC9122319 35594348

[15] H. Fan and J. P. Gong , “Bioinspired Underwater Adhesives,” Advanced Materials 33 (2021): 2102983, 10.1002/adma.202102983. 34532910

[16] K. Yuan , C. He , Z. Che , et al., “Press‐to‐Seal Hydrogel Patches With Spatiotemporally Controllable Blood Repulsion for Acute Hemostasis and Enhanced Healing of Deep Incisive Wounds,” Advanced Materials 38 (2026): 16490, 10.1002/adma.202516490. 41361946

[17] B. Jia , X. Zhao , X. Wan , Z. Wu , Y. Wu , and H. Huang , “Biofunctional and Interface‐Engineered Hydrogels for Advanced Tissue Engineering,” Advanced Healthcare Materials 14 (2025): 2502146, 10.1002/adhm.202502146. 40817676

[18] Y. Zhang , Y. Pan , R. Chang , et al., “Advancing Homogeneous Networking Principles For The Development of Fatigue‐resistant, Low‐swelling and Sprayable Hydrogels For Sealing Wet, Dynamic and Concealed Wounds in Vivo,” Bioactive Materials 34 (2023): 150–163, 10.1016/j.bioactmat.2023.12.002. 38225944 PMC10788230

[19] X. Zhao , J. Luo , Y. Huang , et al., “Injectable Antiswelling and High‐Strength Bioactive Hydrogels With a Wet Adhesion and Rapid Gelling Process to Promote Sutureless Wound Closure and Scar‐Free Repair of Infectious Wounds,” ACS Nano 17 (2023): 22015–22034, 10.1021/acsnano.3c08625. 37862553

[20] Z. Chen , H. Wu , H. Wang , et al., “An Injectable Anti‐Microbial and Adhesive Hydrogel for the Effective Noncompressible Visceral Hemostasis and Wound Repair,” Materials Science and Engineering: C 129 (2021): 112422, 10.1016/j.msec.2021.112422. 34579930

[21] J. He , Z. Zhang , Y. Yang , et al., “Injectable Self‐Healing Adhesive pH‐Responsive Hydrogels Accelerate Gastric Hemostasis and Wound Healing,” Nano‐Micro Letters 13 (2021): 80, 10.1007/s40820-020-00585-0. 34138263 PMC8187506

[22] W. Zhang , S. Song , J. Huang , and Z. Zhang , “An Injectable, Robust Double Network Adhesive Hydrogel for Efficient, Real‐Time Hemostatic Sealing,” Chemical Engineering Journal 476 (2023): 146244, 10.1016/j.cej.2023.146244.

[23] Q. Bai , Y. Hong , Y. Huang , et al., “Body Fluid‐Triggered Adhesive Hydrogels for Emergency Hemostasis and Alveolar Regeneration,” Cell Biomaterials 2 (2025): 100250, 10.1016/j.celbio.2025.100250.

[24] S. Li , L. Zhi , Q. Chen , W. Zhao , and C. Zhao , “Reversibly Adhesive, Anti‐Swelling, and Antibacterial Hydrogels for Tooth‐Extraction Wound Healing,” Advanced Healthcare Materials 13 (2024): 2400089, 10.1002/adhm.202400089. 38354105

[25] Z. Deng , L. Shen , Q. Cheng , Y. Li , Q. Liu , and X. Zhao , “Anti‐Swelling Dual‐Network Zwitterionic Conductive Hydrogels for Flexible Human Activity Sensing,” Polymers 17 (2025): 2230, 10.3390/polym17162230. 40871177 PMC12389484

[26] Z. Ji , D. Gong , M. Zhu , et al., “Mussel‐Inspired Adhesive and Anti‐Swelling Hydrogels for Underwater Strain Sensing,” Soft Matter 20 (2024): 629–639, 10.1039/D3SM01503C. 38163997

[27] G. Singh and A. Chanda , “Mechanical Properties of Whole‐Body Soft Human Tissues: A Review,” Biomedical Materials 16 (2021): 062004, 10.1088/1748-605X/ac2b7a. 34587593

[28] H. Ren , Z. Zhang , X. Cheng , Z. Zou , X. Chen , and C. He , “Injectable, Self‐Healing Hydrogel Adhesives with Firm Tissue Adhesion and on‐Demand Biodegradation for Sutureless Wound Closure,” Science Advances 9 (2023): adh4327, 10.1126/sciadv.adh4327. PMC10431709 37585520

[29] L. Zhou , C. Dai , L. Fan , et al., “Injectable Self‐Healing Natural Biopolymer‐Based Hydrogel Adhesive with Thermoresponsive Reversible Adhesion for Minimally Invasive Surgery,” Advanced Functional Materials 31 (2021): 2007457, 10.1002/adfm.202007457.

[30] X. Zhao , Y. Huang , Z. Li , et al., “Injectable Self‐Expanding/Self‐Propelling Hydrogel Adhesive with Procoagulant Activity and Rapid Gelation for Lethal Massive Hemorrhage Management,” Advanced Materials 36 (2024): 2308701, 10.1002/adma.202308701. 37971104

[31] Q. Li , W. Song , J. Li , et al., “Bioinspired Super‐strong Aqueous Synthetic Tissue Adhesives,” Matter 5 (2022): 933–956, 10.1016/j.matt.2021.12.018. 35252844 PMC8896806

[32] Y. Hong , F. Zhou , Y. Hua , et al., “A Strongly Adhesive Hemostatic Hydrogel For The Repair Of Arterial and Heart Bleeds,” Nature Communications 10 (2019): 2060, 10.1038/s41467-019-10004-7. PMC6517429 31089131

[33] W. Yang , X. Kang , X. Gao , et al., “Biomimetic Natural Biopolymer‐Based Wet‐Tissue Adhesive for Tough Adhesion, Seamless Sealed, Emergency/Nonpressing Hemostasis, and Promoted Wound Healing,” Advanced Functional Materials 33 (2023): 2211340, 10.1002/adfm.202211340.

[34] A. S. Cifu and A. M. Davis , “Prevention, Detection, Evaluation, and Management of High Blood Pressure in Adults,” JAMA 318 (2017): 2132, 10.1001/jama.2017.18706. 29159416

[35] Z. Yu , D. Zhao , Y. Zhang , et al., “Uncovering Novel Therapeutic Clues For Hypercoagulable Active Ulcerative Colitis: Novel Findings From Old Data,” Gastroenterology Report 12 (2024): goae105, 10.1093/gastro/goae105. 39735422 PMC11681937

[36] H. Wang , J. Cheng , F. Sun , et al., “A Super Tough, Rapidly Biodegradable, Ultrafast Hemostatic Bioglue,” Advanced Materials 35 (2023): 2208622, 10.1002/adma.202208622. 36579739

[37] N. Zhang , R. Yao , J. Guo , J. He , G. Meng , and F. Wu , “Modulation of Osteogenic and Haemostatic Activities by Tuning Cationicity of Genipin‐Crosslinked Chitosan Hydrogels,” Colloids and Surfaces B: Biointerfaces 166 (2018): 29–36, 10.1016/j.colsurfb.2018.02.056. 29529506

[38] A. A. Alali and A. N. Barkun , “An Update on the Management of Non‐Variceal Upper Gastrointestinal Bleeding,” Gastroenterology Report 11 (2023): goad011, 10.1093/gastro/goad011. 36949934 PMC10027415

[39] B. Xue , Z. Xu , L. Li , et al., “Hydrogels with Programmed Spatiotemporal Mechanical Cues for Stem Cell‐Assisted Bone Regeneration,” Nature Communications 16 (2025): 3633, 10.1038/s41467-025-59016-6. PMC12003706 40240370

[40] K. Zhao , L. Qi , Q. Li , Y. Wang , C. Qian , and Z. Shi , “Self‐Absorbing Multilayer Skin‐Like Composite with Phyllostachys Nigra Polysaccharides Promotes Wound Healing,” Advanced Composites and Hybrid Materials 7 (2024): 225, 10.1007/s42114-024-01018-x.

[41] Q. Guan , S. Hou , K. Wang , et al., “Micropore Structure Engineering of Injectable Granular Hydrogels via Controlled Liquid‐Liquid Phase Separation Facilitates Regenerative Wound Healing in Mice and Pigs,” Biomaterials 318 (2025): 123192, 10.1016/j.biomaterials.2025.123192. 39965423

