# Supplementary material for: Injectable Hydrogel with Rapid Coagulation, Low Swelling, and High Burst Pressure Tolerance Properties for Long‐Term Management of Bleeding Wound
Source: Adv Sci (Weinh). 2026 Apr 24;13(38):e75329. doi: 10.1002/advs.75329 (PMC13335459; doi:10.1002/advs.75329)
Supplement: Supplementary file 1 — Supporting File 1: advs75329‐sup‐0001‐SuppMat.docx. [file ADVS-13-e75329-s007.docx]

Supporting Information

**Injectable Hydrogel with Rapid Coagulation, Low Swelling, and High Burst Pressure Tolerance Properties for Long-Term Management of Bleeding Wound**

Yang Ouyang^1^, Wenfeng Qiu^2^, Weiwen Liang^3^, Xin Zhang^4^, Weijie Liu^5^, Zixin Chen^1^, Yifei Li^1^, Hui Wang^1^, Youchen Tang^6,*^, Min Li^7,*^, Rongkang Huang^1^, Zijian Chen^1,*^, Binghua Ma^8*^

^1^Colorectal Surgery Unit III, Guangdong Institute of Gastroenterology, Biomedical Innovation Center, Key Laboratory of Human Microbiome and Chronic Diseases, Sun Yat-sen University, Ministry of Education, Guangdong Provincial Key Laboratory of Colorectal and Pelvic Floor Diseases, The Sixth Affiliated Hospital, Sun Yat-sen University, Guangzhou, P. R. China

^2^Colorectal Surgery Unit V, Guangdong Institute of Gastroenterology, Biomedical Innovation Center, Key Laboratory of Human Microbiome and Chronic Diseases, Sun Yat-sen University, Ministry of Education, Guangdong Provincial Key Laboratory of Colorectal and Pelvic Floor Diseases, The Sixth Affiliated Hospital, Sun Yat-sen University, Guangzhou, P. R. China

^3^Department of General surgery (Thyroid Surgery), Guangdong Provincial Key Laboratory of Malignant Tumor Epigenetics and Gene Regulation, Medical Research Center, Sun Yat-Sen Memorial Hospital, Sun Yat-Sen University, Guangzhou, P.R. China

^4^Naval Medical Service Training Base, Naval Medical University, Shanghai, P.R. China

^5^PCFM Lab, School of chemistry, Sun Yat-sen University, Guangzhou, P. R. China

^6^The Eighth Affiliated Hospital, Sun Yat-sen University, Shenzhen, P. R. China

^7^Department of Gastrointestinal Surgery, The Affiliated Dongguan Songshan Lake Central Hospital, Guangdong Medical University, Dongguan, P. R. China

^8^Translational Medicine Research Center, Naval Medical University, Shanghai, P. R. China

***Materials***

CmCS (degree of carboxymethylation 80%, viscosity 10–80 mPa·s) was purchased from Meilunbio (China). Tetra-PEG-SS (M*w* 10 kDa) was obtained from SINOPEG (China). 1-(3-Dimethylaminopropyl)-3-ethylcarbodiimide (EDC), ethylenediamine (ED), Cy5.5 NHS ester，and hydrochloric acid (HCl)were purchased from Macklin (China). Dialysis membranes (molecular weight cutoff 3500 Da) were purchased from YuanYe Biotechnology (China). Dulbecco's Modified Eagle Medium (DMEM), fetal bovine serum (FBS), penicillin–streptomycin solution (PSS), and trypsin-ethylenediaminetetraacetic acid (trypsin-EDTA) were purchased from Thermo Fisher Scientific (USA). Cell Counting Kit-8 (CCK-8) and calcein acetoxymethyl ester (calcein AM) staining reagent were purchased from Solarbio Science and Technology Co., Ltd. (China). Phosphate-buffered saline (PBS), primary antibodies against interleukin-6 (anti-IL-6,) and Anti-CD68 were purchased from Wuhan Servicebio Technology Co., Ltd. (China). Commercial porcine Fibrin gel was purchased from Guangzhou Bioseal Biotech Co., Ltd. (China), and thrombin lyophilized powder purchased from Hunan Yige Pharmaceutical Co., Ltd. (China). All solvents and chemicals were purchased from commercial sources and used without further purification. Male Sprague–Dawley rats (8 weeks old, 200–300 g), male New Zealand rabbits (2.0-2.5 kg), and female Landrace pigs (approximately 40 kg) were obtained from the Laboratory Animal Center of Sun Yat-sen University (China), Guangzhou Longguixingke Animal Center (China), and Jiangxi Yinshe Biotechnology Co., Ltd. (China), respectively. The intervention on rats and rabbits was reviewed and approved by the Institutional Animal Care and Use Committee of Sun Yat-sen University (SYSU-IACUC-2025-002759). All procedures involving female Landrace pigs was reviewed and approved by the Institutional Animal Care and Use Committee of Yinshe Medical Technology Co., Ltd (SS-2025-ZSL GP). All experimental procedures strictly adhered to the Laboratory Animal Care and Use Guidelines.

***Synthesis of ACmCS***

CmCS (5 g) was dissolved in PBS (200 mL) under magnetic stirring at room temperature for 12 h until a clear solution was obtained. Ethylenediamine (ED, 32.94 mL, 99%) was then added, and the pH of the mixture was adjusted to 5.0 using 1 M HCl. Subsequently, 1-(3-dimethylaminopropyl)-3-ethylcarbodiimide (EDC, 9.44 g) was introduced, and the reaction was allowed to proceed for 4 h with continuous stirring. The resulting solution was dialyzed against deionized water for 3 days using a dialysis membrane (MWCO 3500 Da), with the water exchanged every 8 h (9 changes total). Finally, the dialyzed solution was frozen at −80°C and lyophilized at −50°C under vacuum (< 10 Pa) for 48 h using a freeze dryer (LGJ-12, Songyuan Huaxing, China) to afford ACmCS as a white fluffy solid.

***Preparation of PACmC Hydrogel***

ACmCS and Tetra-PEG-SS were dissolved in deionized water to obtain precursor solutions of 3–7 wt% and 15 wt%, respectively. PACmC hydrogel was formed by rapidly mixing equal volumes of the two precursors using a dual-barrel syringe at room temperature.

***Fourier Transform Infrared (FTIR) Spectroscopy***

The chemical structure and functional groups of ACmCS were characterized using a FTIR spectroscope (Nicolet iS50, Thermo Fisher Scientific, USA).

***Mie scattering***

The self-assembly capability of ACmCS into particles in solution was observed via Mie scattering by illuminating the solution with a laser pointer.

***Zeta Potential and Particle Size Analysis***

The charge and particle size distribution of ACmCS self-assembled particles were determined using a zeta potential and particle size analyzer (BI-PALS, Brookhaven Instruments Corporation, USA).

***Transmission Electron Microscopy (TEM)***

The microstructure of ACmCS self-assembled particles was observed by TEM (Talos F200X G2, FEI/Thermo Fisher Scientific, USA) operated at an accelerating voltage of 200 kV.

***Scanning Electron Microscopy (SEM)***

The microstructure of PACmC hydrogels was characterized by SEM (S-4800, Hitachi, Japan). The freeze-dried samples were mounted on aluminum stubs with conductive adhesive tape and sputter-coated with gold (sputtering current 10 mA, duration 50 s, repeated twice) before observation. SEM images were acquired at an accelerating voltage of 10.0 kV.

***Rheology***

Oscillatory frequency sweep and time sweep tests were performed using a rotational rheometer (Kinexus Pro+, Malvern, UK) equipped with a DIN standard C25 coaxial cylinder measurement system. Rheological and viscosity measurements of PACmC were carried out at a controlled temperature of 25 °C. Frequency sweep tests were conducted at a strain amplitude of 1.0% over a frequency range of 0.1 to 10.0 Hz.In addition, time sweep tests were performed by measuring the storage modulus (G′) and loss modulus (G″) to monitor the sol-gel transition of PACmC after injection. During the test, the frequency and shear strain were maintained at 1 Hz and 1.0%, respectively. ACmCS colloidal dispersion was first added into the coaxial cylinder, followed by the introduction of Tetra-PEG-SS solution at the 6th second.

***Swelling Ratio Measurement***

The swelling behavior of PC and PACmC hydrogels was evaluated by incubating the samples in PBS at 37°C. The hydrogels were prepared as described previously and allowed to undergo complete gelation. The initial weight (W₀) of each sample was recorded before placing it in a sealed container and storing it in an incubator at 37°C. At predetermined time points (0, 2, 12, 24, 48, 96, and 168 h), the samples were carefully removed from PBS, gently blotted with filter paper to remove surface moisture, and immediately weighed to obtain the wet weight (*W*). The swelling ratio was calculated using the following equation:

$$\text{S}\text{welling ratio}\text{ }=\frac{(W - W₀)}{W₀}\times100\%$$

where *W* represents the wet weight at a given time and W₀ is the initial weight. After weighing, each sample was returned to PBS to continue incubation.

***Mechanical Testing***

All mechanical tests were performed at room temperature. (1) Adhesive shear strength: The shear adhesion of Fibrin gel, PC, and PACmC hydrogels was evaluated via a lap-shear test using a universal testing machine (WD-5A, Guangzhou Testing Instrument Factory, China) equipped with a 50 N load cell. Fresh porcine skin strips were overlapped with a 1 cm × 1 cm adhesion area. After applying the hydrogel and gelling for 10 min, samples were pulled apart until failure. The adhesive shear strength was calculated as the maximum load divided by the adhesion area. (2) Compression test: Unconfined compression tests were conducted on cylindrical hydrogel samples (diameter: 7 mm, height: 10 mm) using the same universal testing machine. Samples were compressed at a rate of 10 mm·min⁻¹ until rupture or 80% strain was reached.

***Burst Pressure Measurement***

The sealing capability was evaluated according to the standard burst pressure test (ASTM F2392-04). A circular defect (diameter: 3 mm) was created in fresh porcine skin and sealed with the hydrogel (patch diameter: 15 mm). After gelling for 10 min, the sealed sample was mounted in a custom fixture. Deionized water was infused through the defect at 1.0 mL· min⁻¹ using a syringe pump (LD-P01-1A, Longer Pump, China), while pressure was monitored with a digital gauge (HT-1891, XINTEST, China). The burst pressure was recorded as the maximum pressure at seal failure. Tests were performed in triplicate.

***Sealing Performance Assessment***

The sealing capability of PACmC hydrogel was evaluated on three distinct porcine tissues under high-pressure conditions. (i) Liquid Sealing on Perforated Aorta: A circular defect (diameter: approximately 2 mm) was created on a porcine aorta segment using a puncture needle. PACmC hydrogel precursor solution was applied to cover the defect (coverage diameter: approximately 15 mm) and gelled for 10 min at room temperature. One end of the aorta was sealed and connected to a digital pressure gauge (HT-1891, XINTEST, China), while the other end was connected to a syringe pump (LD-P01-1A, Longer Pump, China). Red ink was continuously infused until seal failure, and the maximum pressure recorded at the point of leakage was used to quantify the sealing performance. (ii) Liquid Sealing on Damaged Heart: A linear incision (length: approximately 10 mm) was made on a porcine heart surface. After applying the hydrogel precursor to cover the wound (coverage diameter: approximately 30 mm) and allowing it to cure for 10 min, red ink was injected into the heart chamber. Sealing effectiveness was assessed by visual inspection for leakage. (iii) Gas Sealing on Incised Lung: A circular defect (diameter: approximately 10 mm) was created on porcine lung tissue. Following hydrogel application and curing (coverage diameter: approximately 20 mm), air was inflated into the lung via a tracheal tube to supraphysiological pressures. The sealed tissue was submerged in water, and sealing performance was evaluated by monitoring bubble formation at the wound site.

***Coagulation Assay***

PACmC hydrogel, fibrin gel, and a blank control were coated onto the bottom of consecutive wells in a 48-well plate at a volume of 150 μL per well. A mixture of anticoagulated rabbit blood and calcium chloride (375 μL) at a ratio of 10:1 was gently pipetted onto the surface of each sample to ensure thorough infiltration. The plate was then incubated at 37°C. At predetermined time points, the supernatant was collected to measure its absorbance, yielding the optical density value at the corresponding time point (*ODₓ*). Whole blood without calcium chloride was used to obtain the baseline absorbance (*OD₀*). These values were used to calculate the clotting index. To terminate the reaction, 2 mL of ultrapure water was added, and each well was washed with PBS to completely remove non-clotted blood. The time required for the formation of a stable clot in each well was recorded as the clotting time. The clotting index was calculated using the following formula:

$$\text{Clotting index }\left( \% \right)=\frac{ODₓ}{OD_{\text{0}}}\times100\%$$

***Hemolysis Assay***

Red blood cells were collected from whole blood samples of New Zealand rabbits by centrifugation. Briefly, 300 μL of whole blood was centrifuged at 1500 rpm for 10 min, and the obtained red blood cells were washed three times with PBS. The resulting red blood cell pellet was diluted with normal saline to a final volume of 6000 μL to obtain a red blood cell suspension. For the assay, 40 μL of the sterilized sample was added to a well plate and allowed to form a gel in situ. Subsequently, 60 mL of PBS and 200 μL of the red blood cell suspension were added. Positive and negative controls were set as follows: negative control consisted of 60 mL of PBS and 200 μL of red blood cell suspension; positive control consisted of 60 μL of Triton X-100 solution and 200 μL of red blood cell suspension. The samples were incubated at 37°C for 3 h, followed by centrifugation at 2000 rpm for 10 min. Then, 100 μL of the supernatant was collected, and its absorbance was measured at 570 nm and 545 nm using a microplate reader. The hemolysis rate was calculated using the following formula:

$$\text{Hemolysis }\left( \% \right)=\frac{\left( OD_{sample}-\text{ }OD_{\text{negative}} \right)}{\left( OD_{\text{positive}}\text{ }-\text{ }OD_{\text{negative}} \right)}\times100\%$$

***Cytocompatibility Assessment***

1.0 mL of the hydrogel sample was mixed with 5.0 mL of DMEM solution and placed in a 37°C incubator for 24 h. Then, the above conditioned culture medium was obtained by a 0.22 μm bacterial filter for further use. 3 × 10³ L929 fibroblasts were seeded in 96-well plates with 100 μL of DMEM solution, 10% FBS, and 1% penicillin−streptomycin, which were incubated overnight under 5% CO_2_ at 37°C. 100 μL of the conditioned culture medium was added to each well of the test group, while 100 μL of DMEM solution was added to each well of the control group. At 24, 48, and 72 h after incubation, 100 μL of the 10% CCK8 assay solution was added accordingly to each well of both groups under dark conditions. After 1 h, cell proliferation was determined by measuring the fluorescence absorbance at a wavelength of 450 nm. Optical density (OD) values were measured using a Multifunctional Microplate Reader (SpectraMaxiD3, Molecular Devices, USA). Additionally, 100 μL of the 1% calcein-AM solution was added to each well of both groups under dark conditions. After 15 min, stained L929 fibroblast cells were observed under an inverted fluorescence microscope (AE31E, Motic, China), with observation of the live cells.

***In Vivo Biocompatibility Assessment***

Male Sprague–Dawley rats were anesthetized by intraperitoneal injection of 3% sodium pentobarbital. A dorsal skin incision was made, and 200 μL of PACmC hydrogel was implanted into the subcutaneous tissue. The control group received a subcutaneous injection of 200 μL of normal saline. All incisions were subsequently sutured. At designated time points post-implantation, tissue samples were collected, fixed, paraffin-embedded, and sectioned for HE staining to evaluate morphological changes. Immunohistochemical staining was performed to detect the expression of inflammatory markers, including IL-6 and TNF-α.

***In Vivo Retention Assessment***

A mixture was prepared by combining 1 mg of Cy5.5 NHS ester with 1.0 mL of PACmC hydrogel. Then, 200 µL of the mixture was injected onto the liver surface of each rat. Rat images were acquired using a small animal in vivo imaging system (IVIS Lumina, PerkinElmer, USA) on day 0, 1, 3, 5, 7, 14, 21, and 28 postinjection. The corresponding fluorescence intensity was quantified via the instrument's software to evaluate the in vivo retention of PACmC hydrogel.

***Rabbit Liver and Spleen Injury Hemostasis Models***

The hemostasis models were established in male New Zealand rabbits. After anesthesia via intravenous injection of 3% pentobarbital sodium through the marginal ear vein, a midline laparotomy was performed to expose the liver or spleen. For liver hemostasis, a circular wound (diameter: 10 mm, depth: 5 mm) was created on the liver surface using surgical scissors. For spleen hemostasis, a linear incision (length: 10 mm, depth: 3 mm) was made. Following wound creation and clearance of the surgical field, hemostasis was performed using one of the following methods: (1) application of PACmC hydrogel to cover the wound, (2) gauze compression, or (3) application of commercial thrombin powder. Hemostatic time was recorded from intervention until the cessation of active bleeding, and blood loss was quantified by weighing the blood-saturated gauze. After achieving hemostasis, the abdominal incision was sutured. Animals were monitored for 14 days, after which a second laparotomy was conducted to assess wound healing. Tissue samples from the injury site were harvested for histological evaluation, including HE, Masson, and CD68 immunohistochemical staining.

***Porcine Liver and Spleen Injury Hemostasis Models***

Female Landrace pigs were used for the hemostasis evaluation, with each animal assigned to either liver or spleen injury hemostasis evaluation. Following anesthesia, a midline laparotomy was performed to expose the target organ. For liver hemostasis, a large rectangular wound (length: 40 mm, width: 10 mm, depth: 5 mm) was created on the liver surface using a surgical scalpel. For spleen hemostasis, a linear incision (length: 30 mm, depth: 5 mm) was made. After clearing the surgical field of blood, PACmC hydrogel was promptly applied to cover the wound. Hemostatic time was recorded from application until bleeding ceased, and total blood loss was quantified by weighing the blood-saturated gauze before and after the procedure. After achieving hemostasis, the abdominal incision was sutured. Pigs were monitored for 14 days, after which a second laparotomy was conducted to evaluate wound healing. Tissue samples from the injury sites were harvested for histological assessment, including HE and Masson staining.

***Statistical Analysis***

All experimental data were expressed as mean ± standard deviation (SD). Statistical analyses were performed using OriginPro 2021 software (OriginLab Corporation, USA). Student's t-test was used for comparisons between two groups. For multiple group comparisons, one-way analysis of variance (ANOVA) followed by Tukey's multiple comparison test was used for data with homogeneous variances, while the Kruskal–Wallis nonparametric test was used for data with heterogeneous variances. Statistical significance indicated as * *p* < 0.05, ** *p* < 0.01, and *** *p* < 0.001.


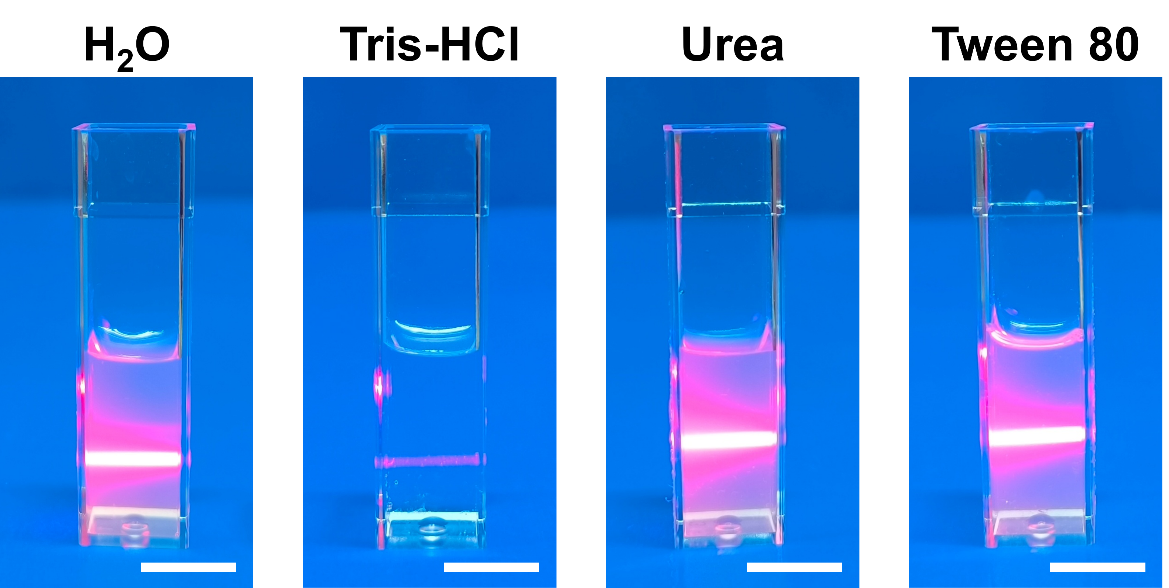


**Figure S1.** Mie scattering of ACmCS in different solutions, such as H_2_O, Tris-HCl (pH 8.5), Urea (0.1%) and Tween-80 (0.1%). Urea and Tween-80 serve as disruptors of hydrogen bonding and hydrophobic interactions, respectively. Scale bars: 10 mm.


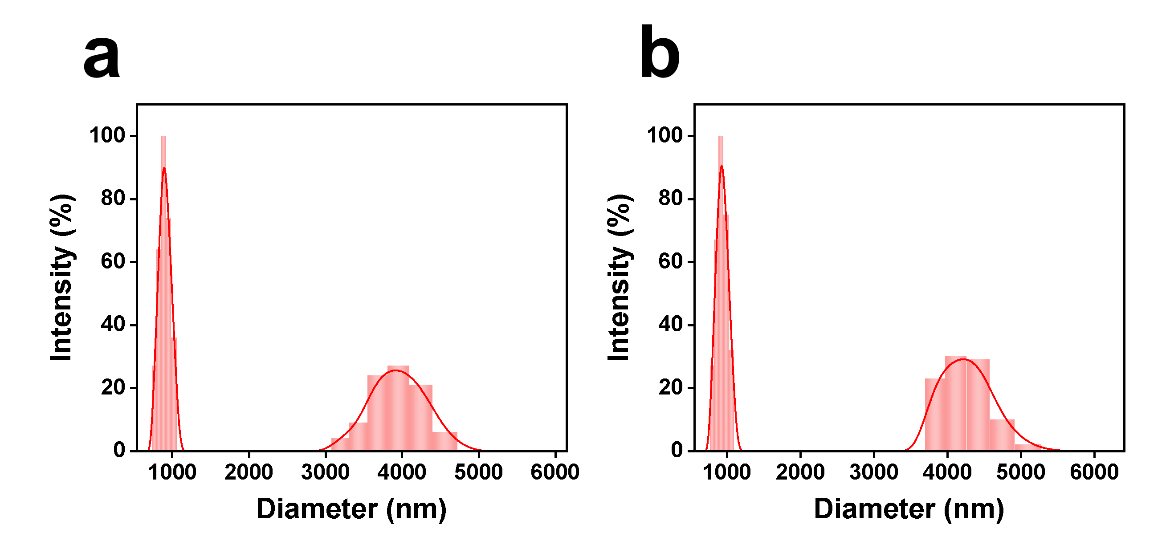


**Figure S2.** Particle size distribution of ACmCS nanoparticles with concentrations of 5 mg·mL^-1^ (a) and 10 mg·mL^-1^ (b) in deionized water.


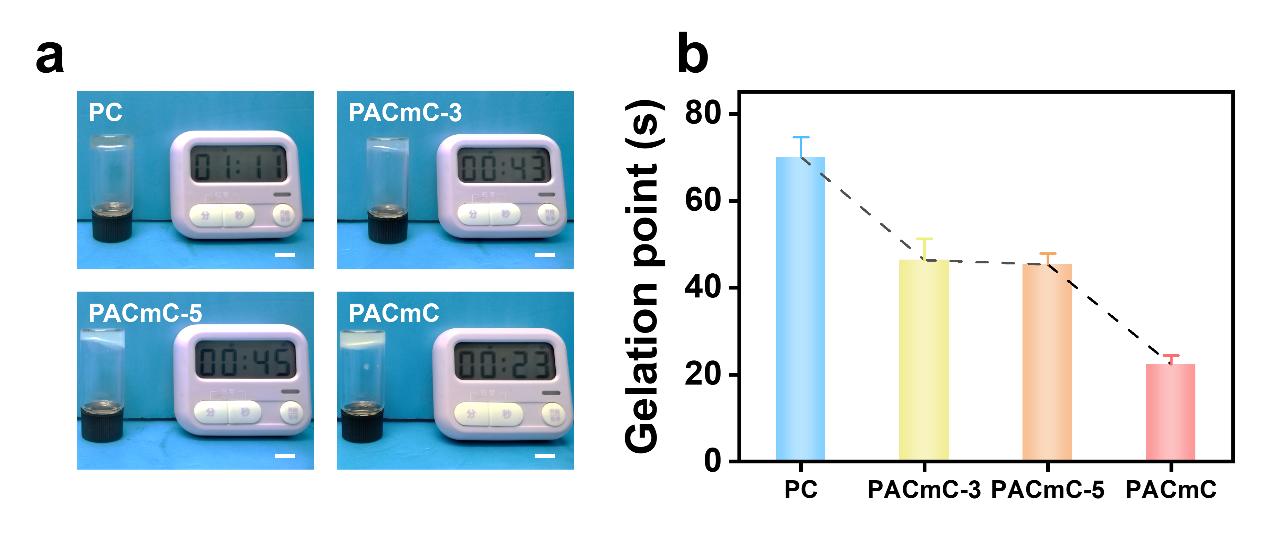


**Figure S3.** Gelation points of PC (prepared from 7 wt% CmCS solution), PACmC-3 (3 wt% ACmCS), PACmC-5 (5 wt% ACmCS), and PACmC (7 wt% ACmCS) hydrogels. Scale bars: 10 mm. The data are the mean ± SD (*n* = 3 independent samples).


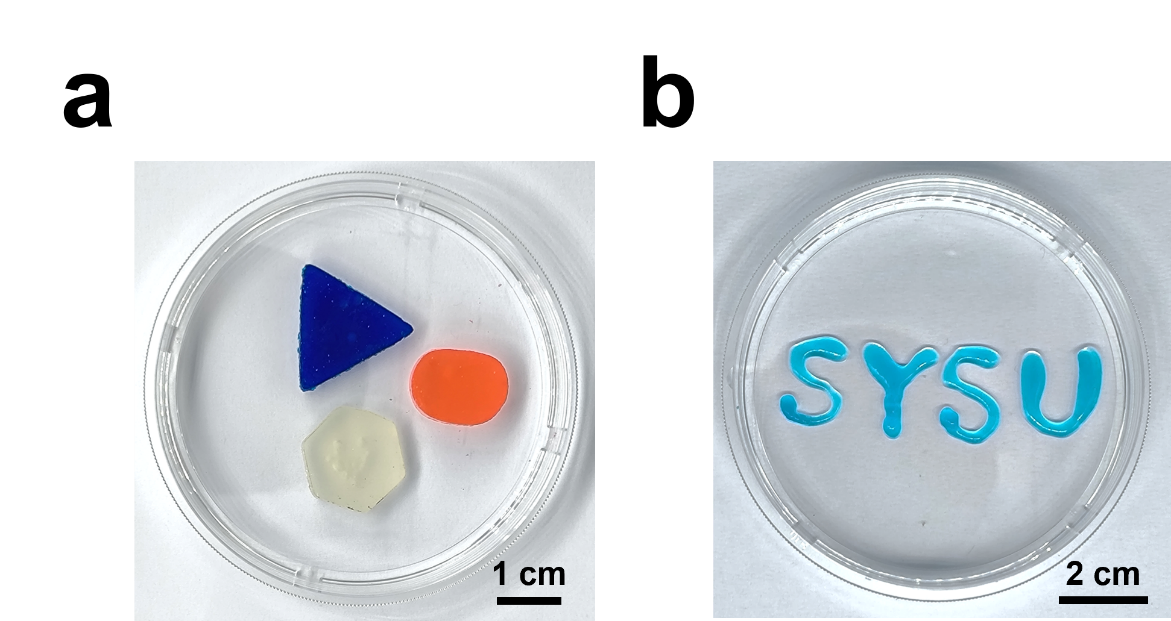


**Figure S4.** a) Digital photo of PACmC hydrogels with different shapes. b) Digital photo of specific letters formed by continuous injection of PACmC hydrogel.


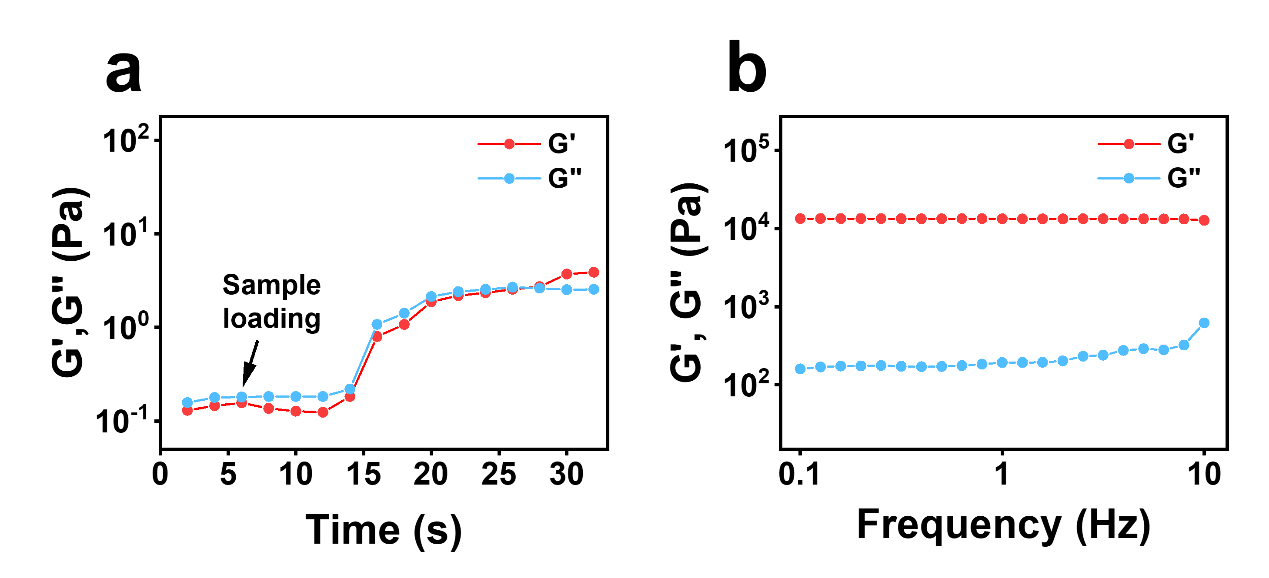


**Figure S5.** a) Oscillatory time sweep of PACmC hydrogel. b) Oscillatory frequency sweep of PACmC hydrogel.


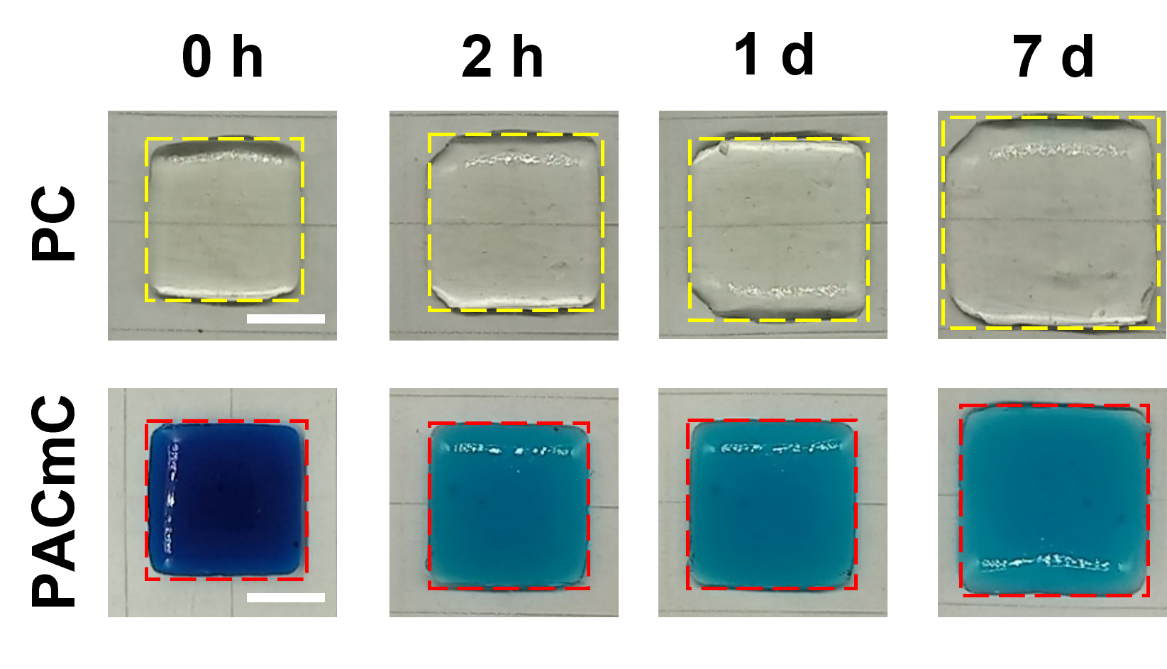


**Figure S6.** Digital photos of PC and PACmC hydrogels before and after immersion in PBS at different time points. Scale bars: 10 mm.

**
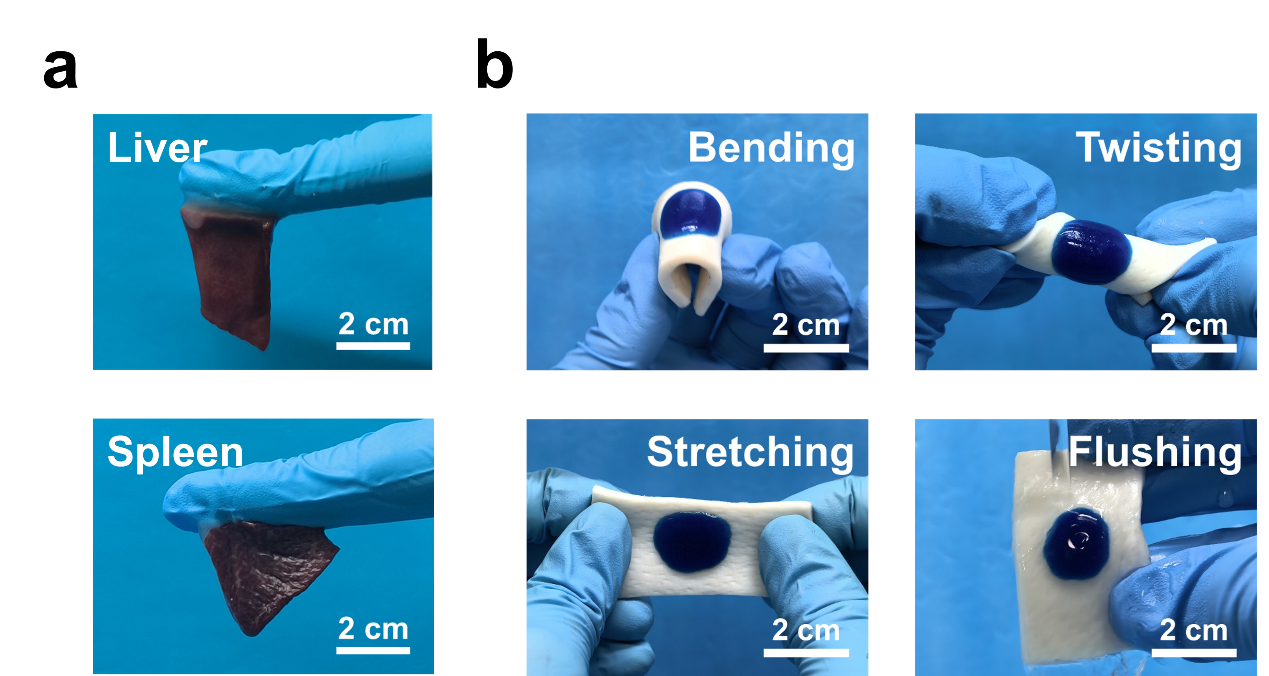
**

**Figure S7.** Adhesion of PACmC hydrogel on porcine liver, spleen, and skin. a) Adhesion on liver and spleen. b) Adhesion on skin and deformation tests.


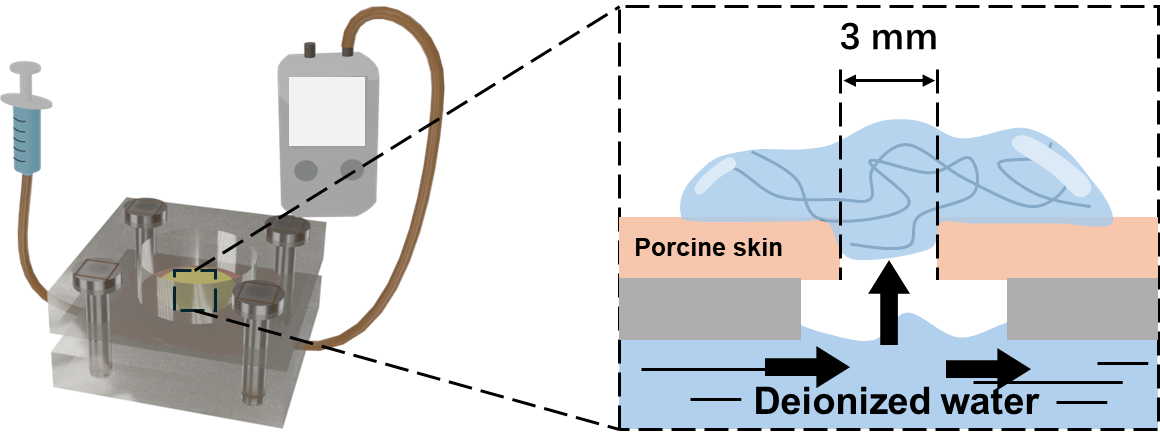


**Figure S8.** Schematic illustration of burst pressure testing.


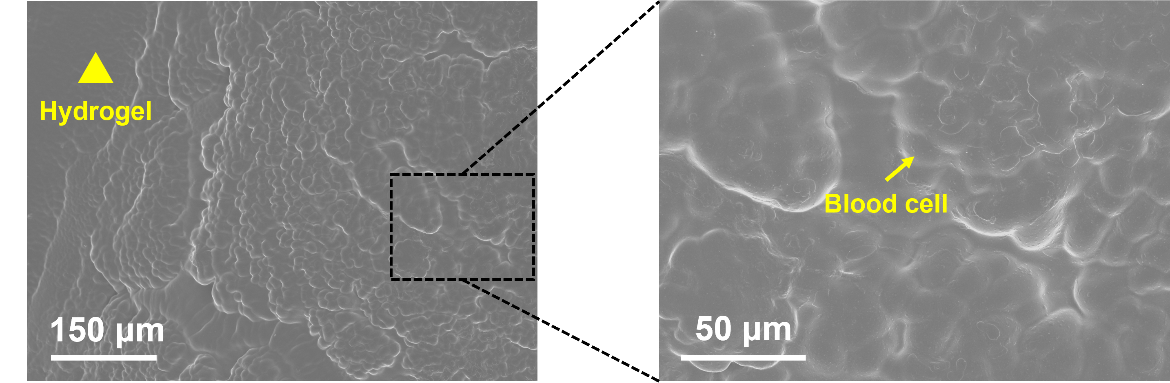


**Figure S9.** Cryo‑SEM images of the blood cells adhered to the PACmC hydrogel surface.


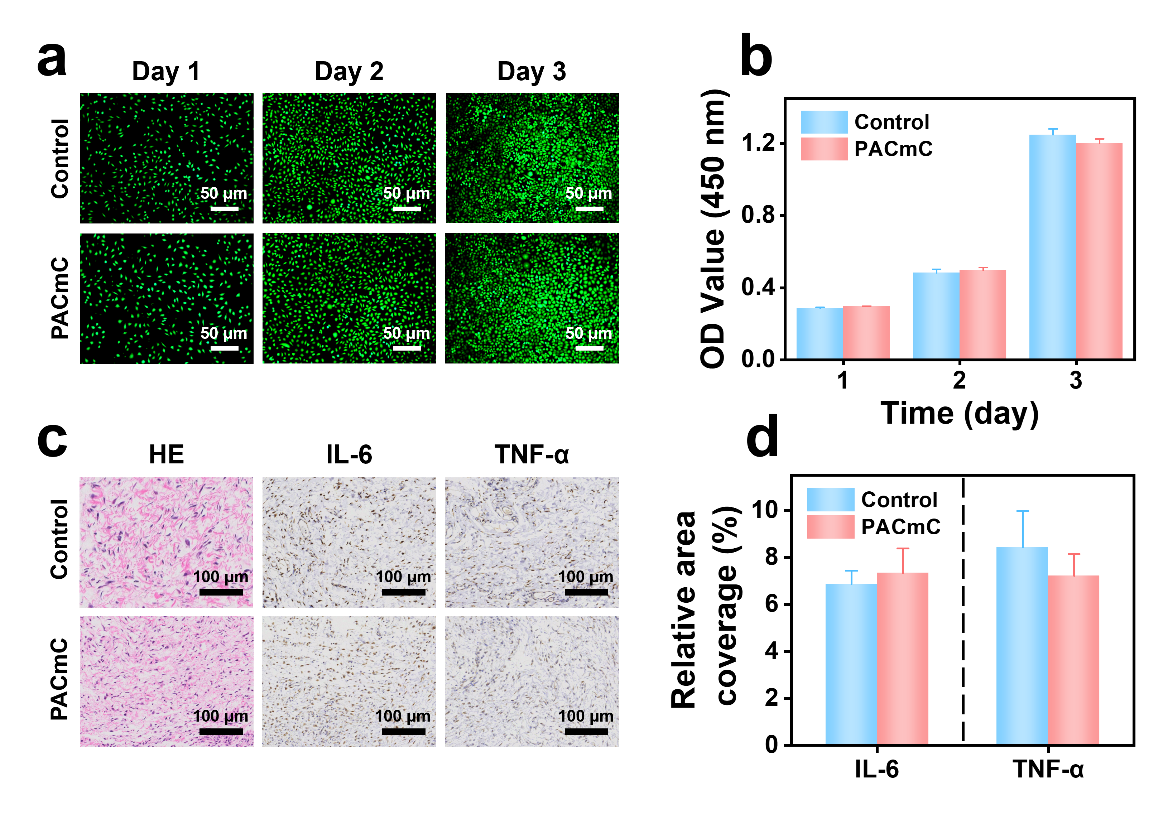


**Figure S10.** Biocompatibility of PACmC hydrogel. a,b) Fluorescence images (a) and CCK-8 assay (b) of L929 ﬁbroblasts cultured on days 1, 2, and 3 in control media (DMEM) and PACmC hydrogel-conditioned media. The data are the mean ± SD (*n* = 3 independent samples). c) Images of HE staining and immunohistochemical staining of IL-6 and TNF-α for the control (200 μL 0.9% physiological saline) and PACmC hydrogel groups after rat subcutaneous implantation for 7 days. d) Quantitative analysis of IL-6 and TNF-α. The data are the mean ± SD (*n* = 3 independent samples).


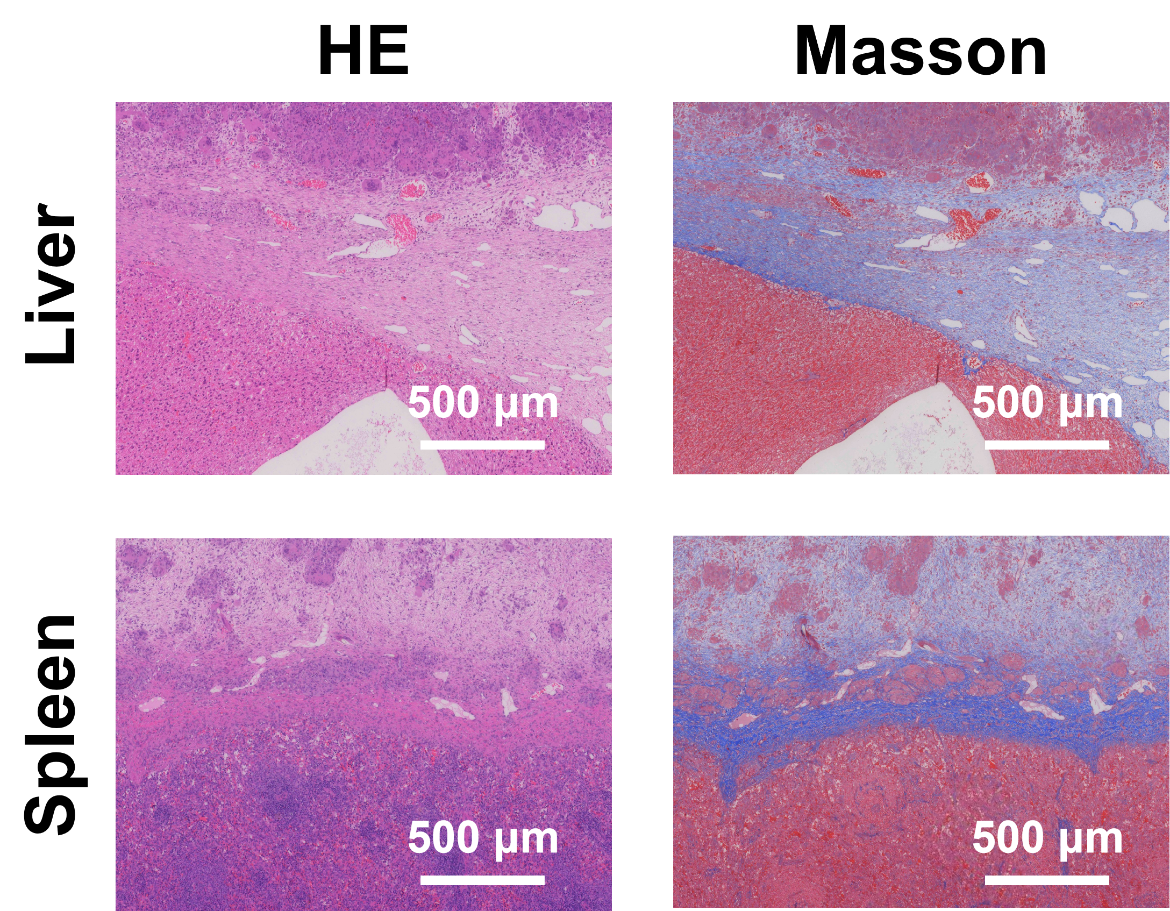


**Figure S11.** HE and Masson staining images of hepatic and splenic injury tissues after the treatment of PACmC hydrogel for 14 days in rabbits.


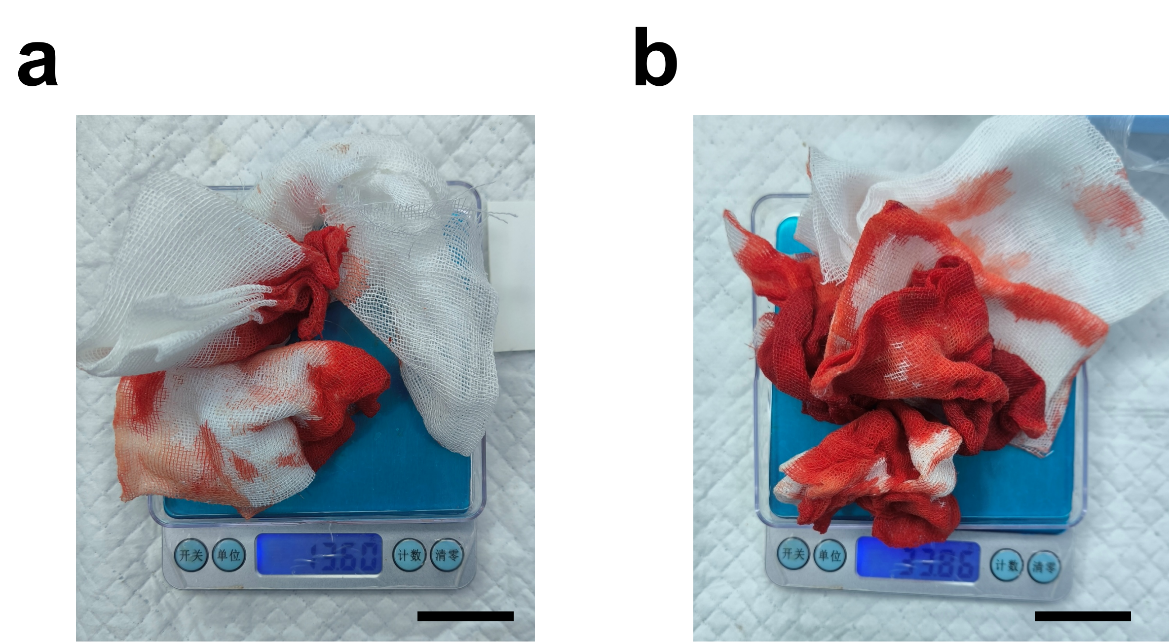


**Figure S12.** Comparison of blood-saturated gauze in hepatic injury model of a pig treated with PACmC hydrogel (a) and suture (b). Scale bars: 30 mm.


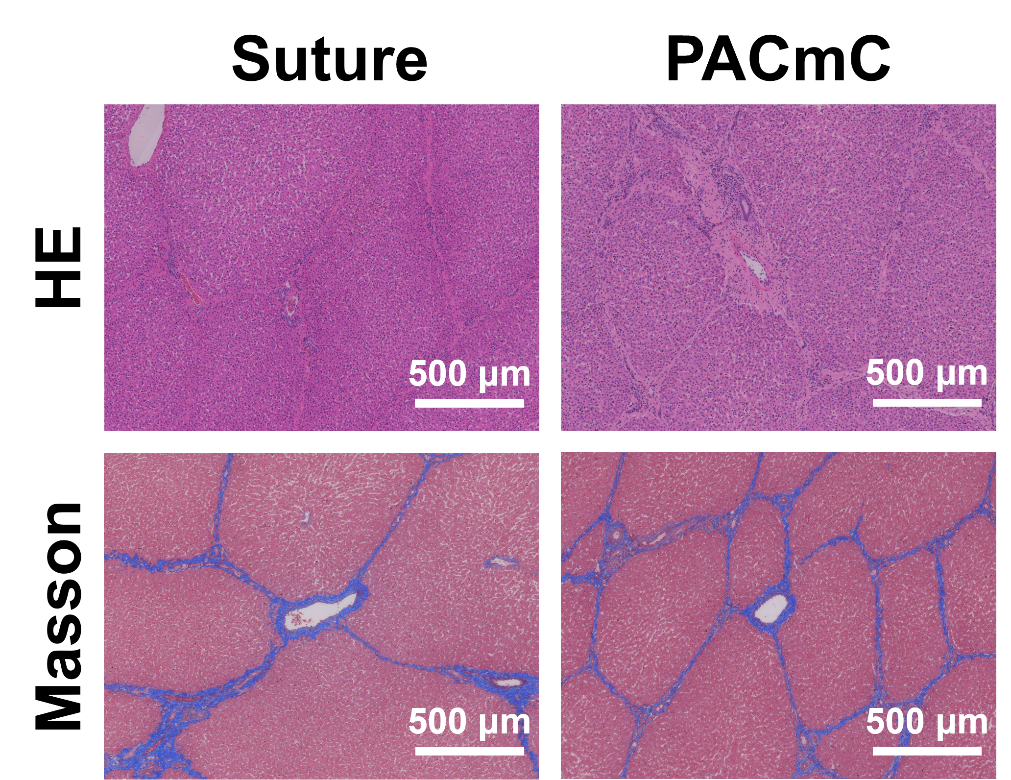


**Figure S13.** HE and Masson staining images of porcine liver interior tissues after the treatment of PACmC hydrogel for 14 days.


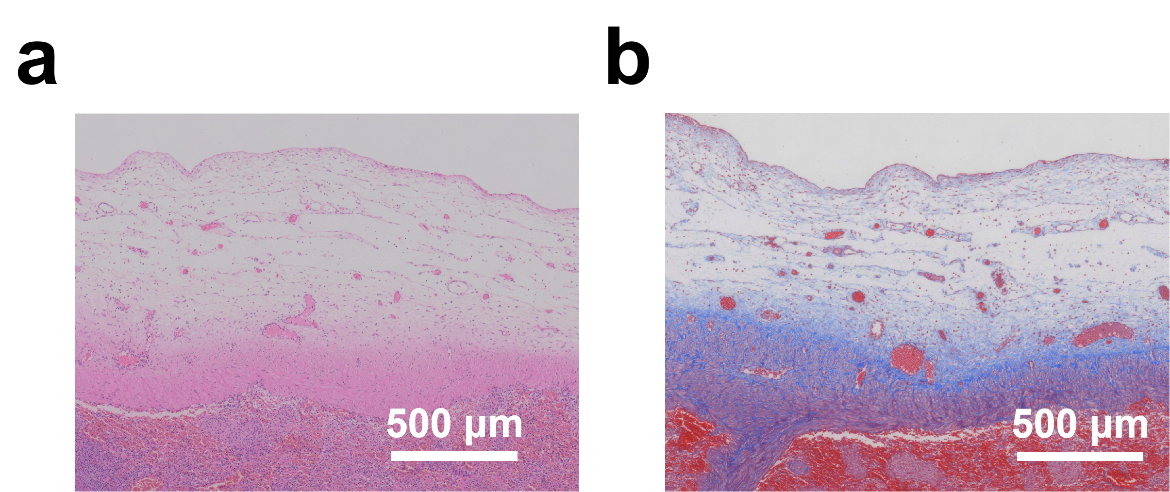


**Figure S14.** HE (a) and Masson (b) staining images of porcine splenic injury tissues after the treatment of PACmC hydrogel for 14 days.

**Table S1.** Comparison of key properties of representative injectable hydrogels.

| **Hydrogel** | **Swelling ratio (%)** | **Burst pressure (mm Hg)** | **Adhesive strength (kPa)** | **Gelation time (s)** | **References** |
| --- | --- | --- | --- | --- | --- |
| **PACmC** | 49.2 | 701 | 46.5 | 22.3 | **This work** |
| **ES gel** | 350 | 450 | 29.4 | 5 | Adv Mater, 2024, 36, 2404811 |
| **CoSt** | — | 153.2 | 62 | <60 | Adv Funct Mater, 2023, 33, 2211340 |
| **HA-PEG** | — | 296.4 | 27.6 | 90 | Sci Adv, 2023, 9, eadh4327 |
| **DNAH** | 150 | 286.5 | 27.5 | ~30−60 | Chem Eng J, 2023, 476, 146244 |
| **Gel-CS** | — | 282 | 30.6 | 180 | Adv Funct Mater, 2021, 31, 2007457 |
| **Matrix gel** | ~4000 | 155−290 | — | <1 | Nat Commun, 2019, 10, 2060 |
| **OD-C/QGQL** | 501 | 235.5 | 10 | 30 | Adv Mater, 2024, 36, 2308701 |
| **Paste** | 800 | ~350 | ~60 | <15 | Nat Biomed Eng, 2021, 5, 1131 |
| **AA/AA-NHS** | 430−670 | — | 6.63–7.96 | <540 | Nano-Micro Lett, 2021, 13, 80 |
| **PAPN gel** | 80 | — | ~18.67 | <10 | Adv Healthcare Mater, 2024, 13, 2400089 |
| **BTTA** | 90 | 351.7 | ~18 | <30 | Cell Biomater, 2025, 2, 100250 |
| **LP** | ~110 | ~136 | ~45−50 | 2 | Bioact Mater, 2024, 34, 150 |
| **PCPD/AS@APF** | 138 | — | ~23.56 | 30−51 | ACS Nano, 2023, 17, 22015 |
| **GelMA/OD/Borax** | ~600 | 165.5 | — | ~12−13 | Mater Sci Eng C, 2021, 129, 112422 |
